# Correction: Detecting rare diseases in electronic health records using machine learning and knowledge engineering: Case study of acute hepatic porphyria

**DOI:** 10.1371/journal.pone.0238277

**Published:** 2020-08-20

**Authors:** Aaron M. Cohen, Steven Chamberlin, Thomas Deloughery, Michelle Nguyen, Steven Bedrick, Stephen Meninger, John J. Ko, Jigar J. Amin, Alex H. Wei, William Hersh

The ninth author's middle initial is incorrect. The correct name is: Alex H. Wei
