# Supplementary material for: Detecting rare diseases in electronic health records using machine learning and knowledge engineering: Case study of acute hepatic porphyria
Source: PLoS One. 2020 Jul 2;15(7):e0235574. doi: 10.1371/journal.pone.0235574 (PMC7331997; doi:10.1371/journal.pone.0235574)
Supplement: S1 Table — Features are scored by number of occurrances in an individual patient medical record, and then normalized. (DOCX) [file pone.0235574.s001.docx]

**S1 Table.** Final 141 features selected for inclusion in the machine learning model to predict acute hepatic porphyria. Features are scored by number of occurrances in an individual patient medical record, and then normalized.

| **INDEX** | **FEATURE** | **SOURCE DOCUMENTS** | **DESCRIPTION** |
| --- | --- | --- | --- |
| 1 | ABDOMINAL_PAIN_DX_NAME | Encounter Diagnosis, Patient Problem List | Text description of diagnosis code (ICD9) |
| 2 | ABDOMINAL_PAIN_UNSPECIFIED_SITE_DX_NAME | Encounter Diagnosis, Patient Problem List | Text description of diagnosis code (ICD9) |
| 3 | ALTERNATIVE_THERAPY_-_PINEAL_HORMONE_AGENTS_PHARM_SUBCLASS_NAME | Concomittent Medications, Administered Medications, Medications Ordered | Text description of drug subclass |
| 4 | ANALGESIC_OPIOID_OXYCODONE_COMBINATIONS_PHARM_SUBCLASS_NAME | Concomittent Medications, Administered Medications, Medications Ordered | Text description of drug subclass |
| 5 | ANTI-ANXIETY_-_BENZODIAZEPINES_PHARM_CLASS_NAME | Concomittent Medications, Administered Medications, Medications Ordered | Text description of drug class |
| 6 | ANTICONVULSANT_-_GABA_ANALOGS_PHARM_SUBCLASS_NAME | Concomittent Medications, Administered Medications, Medications Ordered | Text description of drug subclass |
| 7 | ANTIEMETIC_-_PHENOTHIAZINES_PHARM_SUBCLASS_NAME | Concomittent Medications, Administered Medications, Medications Ordered | Text description of drug subclass |
| 8 | ANTIHISTAMINE_-_1ST_GENERATION_-_ETHANOLAMINES_PHARM_SUBCLASS_NAME | Concomittent Medications, Administered Medications, Medications Ordered | Text description of drug subclass |
| 9 | ANTIHISTAMINE_-_1ST_GENERATION_-_PHENOTHIAZINES_PHARM_SUBCLASS_NAME | Concomittent Medications, Administered Medications, Medications Ordered | Text description of drug subclass |
| 10 | BASO_#_COMPONENT_NAME | Lab Results | Percent Basophils performed |
| 11 | CALCIUM_REPLACEMENT_PHARM_CLASS_NAME | Concomittent Medications, Administered Medications, Medications Ordered | Text description of drug class |
| 12 | CBC_WITH_DIFFERENTIAL_PROC_NAME | Procedures Ordered | CBC with diff order present |
| 13 | CNSLT0031_PROC_CODE | Procedures Ordered | Code for consult to Gastroenterology |
| 14 | CONSULT_TO_GASTROENTEROLOGY_PROC_NAME | Procedures Ordered | Consult to Gastoenterology ordered |
| 15 | COPD_(CHRONIC_OBSTRUCTIVE_PULMONARY_DISEASE)_(HCC)_DX_NAME | Encounter Diagnosis, Patient Problem List | Text description of diagnosis code (ICD9) |
| 16 | CREATININE_URINE_CONCENTRATION_COMPONENT_NAME | Lab Results | lab result component present |
| 17 | CREATININEUR(REFERRAL)_COMPONENT_NAME | Lab Results | lab result component present |
| 18 | DIFFERENTIAL_PROC_NAME | Procedures Ordered | blood differential order present |
| 19 | DIPHENHYDRAMINE_HCL_GENERIC_NAME_1 | Concomittent Medication, Medications Ordered | Generic name of medication |
| 20 | ELEVATED_WHITE_BLOOD_CELL_COUNT_UNSPECIFIED_DX_ICD10_NAME | Encounter Diagnosis, Patient Problem List | Text description of diagnosis code (ICD10) |
| 21 | EOS_#_COMPONENT_NAME | Lab Results | eosinaphil count lab result present |
| 22 | ESSENTIAL_(PRIMARY)_HYPERTENSION_DX_ICD10_NAME | Encounter Diagnosis, Patient Problem List | Text description of diagnosis code (ICD10) |
| 23 | FERRITIN_SERUM_PROC_NAME | Procedures Ordered | serum ferritin order present |
| 24 | HYDROMORPHONE_HCL_GENERIC_NAME_1 | Concomittent Medication, Medications Ordered | Generic name of medication |
| 25 | LAB00047_PROC_CODE | Procedures Ordered | Plasma lipase procedure ordered |
| 26 | LAB00364_PROC_CODE | Procedures Ordered | Microscopic urine exam ordered |
| 27 | LAB00681_PROC_CODE | Procedures Ordered | CBC with differential ordered |
| 28 | LAB100107_PROC_CODE | Procedures Ordered | Blood differential ordered |
| 29 | LAB100227_PROC_CODE | Procedures Ordered | Urine volume measurement ordered |
| 30 | LAB100882_PROC_CODE | Procedures Ordered | Multi-tube blood draw ordered |
| 31 | LIPASE__(LAB)_COMPONENT_NAME | Lab Results | plasma lipase result component present |
| 32 | LIPASE_PLASMA_PROC_NAME | Procedures Ordered | plasma lipase order present |
| 33 | LYMPHOCYTE_#_COMPONENT_NAME | Lab Results | blood lymphocyte count results present |
| 34 | MAGNESIUM_SALTS_REPLACEMENT_PHARM_CLASS_NAME | Concomittent Medications, Administered Medications, Medications Ordered | Text description of drug class |
| 35 | MELATONIN_GENERIC_NAME_1 | Concomittent Medication, Medications Ordered | Generic name of medication |
| 36 | MINERALS_AND_ELECTROLYTES_-_CALCIUM_REPLACEMENT/VITAMIN_D_COMBINATIONS_PHARM_SUBCLASS_NAME | Concomittent Medications, Administered Medications, Medications Ordered | Text description of drug subclass |
| 37 | MISC_REF_TEST_NAME_COMPONENT_NAME | Lab Results | Special test given with name of test in RESULT_TEXT |
| 38 | MISC_REF_TEST_RESULT_COMPONENT_NAME | Lab Results | Result of special test present |
| 39 | MONOCYTE_#_COMPONENT_NAME | Lab Results | blood monocyte count results present |
| 40 | NAUSEA_WITH_VOMITING_UNSPECIFIED_DX_ICD10_NAME | Encounter Diagnosis, Patient Problem List | Text description of diagnosis code (ICD10) |
| 41 | NEUTROPHIL_#_COMPONENT_NAME | Lab Results | blood neutrophil count results present |
| 42 | NGRAM_0^pramipexole | Notes | Bigram of [token]^[token] found in free text. |
| 43 | NGRAM_0^tablet | Notes | Bigram of [token]^[token] found in free text. |
| 44 | NGRAM_10^olanzapine | Notes | Bigram of [token]^[token] found in free text. |
| 45 | NGRAM_10^tablet | Notes | Bigram of [token]^[token] found in free text. |
| 46 | NGRAM_100^sodium | Notes | Bigram of [token]^[token] found in free text. |
| 47 | NGRAM_4^mg | Notes | Bigram of [token]^[token] found in free text. |
| 48 | NGRAM_4^odt | Notes | Bigram of [token]^[token] found in free text. |
| 49 | NGRAM_90^albuterol | Notes | Bigram of [token]^[token] found in free text. |
| 50 | NGRAM_abdominal | Notes | Unigram of [token] found in free text. |
| 51 | NGRAM_abdominal^pain | Notes | Bigram of [token]^[token] found in free text. |
| 52 | NGRAM_acute | Notes | Unigram of [token] found in free text. |
| 53 | NGRAM_acute^distress | Notes | Bigram of [token]^[token] found in free text. |
| 54 | NGRAM_ambulatory | Notes | Unigram of [token] found in free text. |
| 55 | NGRAM_antibiotics | Notes | Unigram of [token] found in free text. |
| 56 | NGRAM_antibiotics^sulfonamide | Notes | Bigram of [token]^[token] found in free text. |
| 57 | NGRAM_atraumatic | Notes | Unigram of [token] found in free text. |
| 58 | NGRAM_bipolar | Notes | Unigram of [token] found in free text. |
| 59 | NGRAM_cigarettes | Notes | Unigram of [token] found in free text. |
| 60 | NGRAM_compazine | Notes | Unigram of [token] found in free text. |
| 61 | NGRAM_control^pain | Notes | Bigram of [token]^[token] found in free text. |
| 62 | NGRAM_depakote | Notes | Unigram of [token] found in free text. |
| 63 | NGRAM_dilaudid | Notes | Unigram of [token] found in free text. |
| 64 | NGRAM_discharged | Notes | Unigram of [token] found in free text. |
| 65 | NGRAM_disintegrating | Notes | Unigram of [token] found in free text. |
| 66 | NGRAM_docusate | Notes | Unigram of [token] found in free text. |
| 67 | NGRAM_docusate^sodium | Notes | Bigram of [token]^[token] found in free text. |
| 68 | NGRAM_dose^oral | Notes | Bigram of [token]^[token] found in free text. |
| 69 | NGRAM_duloxetine | Notes | Unigram of [token] found in free text. |
| 70 | NGRAM_ed | Notes | Unigram of [token] found in free text. |
| 71 | NGRAM_edisylate] | Notes | Unigram of [token] found in free text. |
| 72 | NGRAM_extended^tablet | Notes | Bigram of [token]^[token] found in free text. |
| 73 | NGRAM_fibromyalgia | Notes | Unigram of [token] found in free text. |
| 74 | NGRAM_flare | Notes | Unigram of [token] found in free text. |
| 75 | NGRAM_flares | Notes | Unigram of [token] found in free text. |
| 76 | NGRAM_focal | Notes | Unigram of [token] found in free text. |
| 77 | NGRAM_gallops | Notes | Unigram of [token] found in free text. |
| 78 | NGRAM_genitourinary | Notes | Unigram of [token] found in free text. |
| 79 | NGRAM_glycol | Notes | Unigram of [token] found in free text. |
| 80 | NGRAM_glycol^polyethylene | Notes | Bigram of [token]^[token] found in free text. |
| 81 | NGRAM_gram | Notes | Unigram of [token] found in free text. |
| 82 | NGRAM_hydromorphone | Notes | Unigram of [token] found in free text. |
| 83 | NGRAM_instructed | Notes | Unigram of [token] found in free text. |
| 84 | NGRAM_iv | Notes | Unigram of [token] found in free text. |
| 85 | NGRAM_latex | Notes | Unigram of [token] found in free text. |
| 86 | NGRAM_magnesium | Notes | Unigram of [token] found in free text. |
| 87 | NGRAM_melatonin | Notes | Unigram of [token] found in free text. |
| 88 | NGRAM_miralax | Notes | Unigram of [token] found in free text. |
| 89 | NGRAM_mouth^needed | Notes | Bigram of [token]^[token] found in free text. |
| 90 | NGRAM_mouth^twelve | Notes | Bigram of [token]^[token] found in free text. |
| 91 | NGRAM_nausea | Notes | Unigram of [token] found in free text. |
| 92 | NGRAM_nausea^vomiting | Notes | Bigram of [token]^[token] found in free text. |
| 93 | NGRAM_odt | Notes | Unigram of [token] found in free text. |
| 94 | NGRAM_odt^ondansetron | Notes | Bigram of [token]^[token] found in free text. |
| 95 | NGRAM_olanzapine | Notes | Unigram of [token] found in free text. |
| 96 | NGRAM_oncology | Notes | Unigram of [token] found in free text. |
| 97 | NGRAM_ondansetron | Notes | Unigram of [token] found in free text. |
| 98 | NGRAM_oral^powder | Notes | Bigram of [token]^[token] found in free text. |
| 99 | NGRAM_oxycodone | Notes | Unigram of [token] found in free text. |
| 100 | NGRAM_pain^severe | Notes | Bigram of [token]^[token] found in free text. |
| 101 | NGRAM_pathology | Notes | Unigram of [token] found in free text. |
| 102 | NGRAM_penicillins | Notes | Unigram of [token] found in free text. |
| 103 | NGRAM_phenergan | Notes | Unigram of [token] found in free text. |
| 104 | NGRAM_polyethylene | Notes | Unigram of [token] found in free text. |
| 105 | NGRAM_powder | Notes | Unigram of [token] found in free text. |
| 106 | NGRAM_pramipexole | Notes | Unigram of [token] found in free text. |
| 107 | NGRAM_propranolol | Notes | Unigram of [token] found in free text. |
| 108 | NGRAM_protocol | Notes | Unigram of [token] found in free text. |
| 109 | NGRAM_psychosis | Notes | Unigram of [token] found in free text. |
| 110 | NGRAM_risperidone | Notes | Unigram of [token] found in free text. |
| 111 | NGRAM_rubs | Notes | Unigram of [token] found in free text. |
| 112 | NGRAM_scoliosis | Notes | Unigram of [token] found in free text. |
| 113 | NGRAM_seroquel | Notes | Unigram of [token] found in free text. |
| 114 | NGRAM_severe | Notes | Unigram of [token] found in free text. |
| 115 | NGRAM_stomach | Notes | Unigram of [token] found in free text. |
| 116 | NGRAM_sulfa | Notes | Unigram of [token] found in free text. |
| 117 | NGRAM_sulfonamide | Notes | Unigram of [token] found in free text. |
| 118 | NGRAM_urine | Notes | Unigram of [token] found in free text. |
| 119 | NGRAM_vicodin | Notes | Unigram of [token] found in free text. |
| 120 | NGRAM_zofran | Notes | Unigram of [token] found in free text. |
| 121 | NORMAL_RANGE_COMPONENT_NAME | Lab Results | Lab test result within normal ranges |
| 122 | OBSTRUCTIVE_SLEEP_APNEA_(ADULT)_(PEDIATRIC)_DX_ICD10_NAME | Encounter Diagnosis, Patient Problem List | Text description of diagnosis code (ICD10) |
| 123 | OBSTRUCTIVE_SLEEP_APNEA_DX_NAME | Encounter Diagnosis, Patient Problem List | Text description of diagnosis code (ICD9) |
| 124 | ONDANSETRON_HCL_GENERIC_NAME_1 | Concomittent Medication, Medications Ordered | Generic name of medication |
| 125 | OXYCODONE_HCL/ACETAMINOPHEN_GENERIC_NAME_1 | Concomittent Medication, Medications Ordered | Generic name of medication |
| 126 | PATHOLOGY_PROC_NAME | Procedures Ordered | Transcribed pathology report present |
| 127 | PELVIC_AND_PERINEAL_PAIN_DX_ICD10_NAME | Encounter Diagnosis, Patient Problem List | Text description of diagnosis code (ICD10) |
| 128 | PINEAL_HORMONE_AGENTS_PHARM_CLASS_NAME | Concomittent Medications, Administered Medications, Medications Ordered | Text description of drug subclass |
| 129 | PROCHLORPERAZINE_EDISYLATE_GENERIC_NAME_1 | Concomittent Medication, Medications Ordered | Generic name of medication |
| 130 | PROMETHAZINE_HCL_GENERIC_NAME_1 | Concomittent Medication, Medications Ordered | Generic name of medication |
| 131 | RADIOLOGY_PROC_NAME | Procedures Ordered | Transcribed radiology report present |
| 132 | RAINBOW_HOLD_TUBE_-_BLUE_TOP_PROC_NAME | Procedures Ordered | Multi-tube blood draw ordered |
| 133 | RESTLESS_LEGS_SYNDROME_DX_ICD10_NAME | Encounter Diagnosis, Patient Problem List | Text description of diagnosis code (ICD10) |
| 134 | TOBACCO_ABUSE_DX_NAME | Encounter Diagnosis, Patient Problem List | Text description of diagnosis code (ICD9) |
| 135 | TRIPLE_P04_CRYSTALS_COMPONENT_NAME | Lab Results | Component of result of lab test |
| 136 | TRNS00039_PROC_CODE | Procedures Ordered | Transcribed pathology report present |
| 137 | TRNS00040_PROC_CODE | Procedures Ordered | Transcribed imaging report present |
| 138 | UNSPECIFIED_ABDOMINAL_PAIN_DX_ICD10_NAME | Encounter Diagnosis, Patient Problem List | Text description of diagnosis code (ICD10) |
| 139 | UNSPECIFIED_ABDOMINAL_PAIN_DX_ICD10_NAME | Encounter Diagnosis, Patient Problem List | Text description of diagnosis code (ICD10) |
| 140 | URINE_MICROSCOPIC_EXAM_PROC_NAME | Lab Results | Name of lab test procedure |
| 141 | VOL(URINE)_PROC_NAME | Lab Results | Name of lab test procedure |
