# Supplementary material for: Detecting rare diseases in electronic health records using machine learning and knowledge engineering: Case study of acute hepatic porphyria
Source: PLoS One. 2020 Jul 2;15(7):e0235574. doi: 10.1371/journal.pone.0235574 (PMC7331997; doi:10.1371/journal.pone.0235574)
Supplement: Attachment — Submitted filename: PONE-D-20-10338_R1_reviewer_comments.pdf [file pone.0235574.s003.pdf]

PLOS ONE

Detecting Rare Diseases in Electronic Health Records Using Machine Learning and Knowledge Engineering: Case Study of Acute Hepatic Porphyria

--Manuscript Draft--

**Manuscript Number:**	PONE-D-20-10338R1
**Article Type:**	Research Article
**Full Title:**	Detecting Rare Diseases in Electronic Health Records Using Machine Learning and Knowledge Engineering: Case Study of Acute Hepatic Porphyria
**Short Title:**	Detecting Rare Diseases Using Machine Learning on EHR Data: Case Study of Acute Hepatic Porphyria
**Corresponding Author:**	Aaron M. Cohen, M.D.<br>Oregon Health & Science University<br>Portland, OR UNITED STATES
**Keywords:**	rare diseases; Acute Hepatic Porphyria; machine learning; Data Science; electronic health record
**Abstract:**	Background: With the growing adoption of the electronic health record (EHR) worldwide over the last decade, new opportunities exist for leveraging EHR data for detection of rare diseases. Rare diseases are often not diagnosed or delayed in diagnosis by clinicians who encounter them infrequently. One such rare disease that may be amenable to EHR-based detection is acute hepatic porphyria (AHP). AHP consists of a family of rare, metabolic diseases characterized by potentially life-threatening acute attacks and, for some patients, chronic debilitating symptoms that negatively impact daily functioning and quality of life. The goal of this study was to apply machine learning and knowledge engineering to a large extract of EHR data to determine whether they could be effective in identifying patients not previously tested for AHP who should receive a proper diagnostic workup for AHP.<br>Methods and Findings: We used an extract of the complete EHR data of 200,000 patients from an academic medical center for up to 10 years longitudinally and enriched it with records from an additional 5,571 patients from the center containing any mention of porphyria in notes, laboratory tests, diagnosis codes, and other parts of the record. After manually reviewing all patients with the ICD-10-CM code E80.21 (Acute intermittent [hepatic] porphyria), we identified 30 patients who were positive cases for our machine learning models, with the rest of the patients used as negative cases. We parsed the record into features, which were scored by frequency of appearance and labeled by the EHR source document. We then carried out a univariate feature analysis, manually choosing features not directly tied to provider attributes or suspicion of the patient having AHP. We next trained on the full dataset, with the best cross-validation performance coming from support vector machine (SVM) algorithm using a radial basis function (RBF) kernel. The trained model was applied back to the full data set and patients were ranked by margin distance. The top 100 ranked negative cases were manually reviewed for symptom complexes similar to AHP, finding four patients where AHP diagnostic testing was likely indicated and 18 patients where AHP diagnostic testing was possibly indicated. From the top 100 ranked cases of patients with mention of porphyria in their record, we identified four patients for whom AHP diagnostic testing was possibly indicated and had not been previously performed. Based solely on the reported prevalence of AHP, we would have expected only 0.002 cases out of the 200 patients manually reviewed.<br>Conclusions: The application of machine learning and knowledge engineering to EHR data may facilitate the diagnosis of rare diseases such as AHP. The only manual modifications to this work were the removal of disease-specific or medical center specific features that might undermine our ability to find new cases. Further work will recommend clinical investigation to identified patients' clinicians, evaluate more patients, assess additional feature selection and machine learning algorithms, and apply this methodology to other rare diseases.
**Order of Authors:**	Aaron M. Cohen, M.D.
	Steven Chamberlin

Powered by Editorial Manager® and ProduXion Manager® from Aries Systems Corporation

	Thomas Deloughery
	Michelle Nguyen
	Steven Bedrick
	Stephen Meninger
	John J. Ko
	Jigar J. Amin
	Alex J. Wei
	William Hersh
**Response to Reviewers:**	Reviewer comments and our responses are given in our response letter and more conveniently formatted than are shown here.<br><br>While this is important background it is not clear if this paragraph is needed in the paper, other than noting the diagnostic/prognostics should rely on biomarker and other lab tests rather than family history. Consider removing, or condensing.<br>This paragraph of text is important to provide the patient disease context for our work, and provides additional clinical and genetic background to orient readers who may not have expertise about this disease, such as informaticians and machine learning researchers. The difficult diagnosis of AHP is in part due to the disease low penetrance and inconsistent appearances in families even though AHP and related diseases are mostly autosomal dominant. We therefore would like to keep the paragraph that is there now, as it really does not substantially lengthen the paper.<br><br>Recommend adding the number of patients with ICD-10 code E80.21.<br>This has been done.<br><br>Unique patients, or unique records/document counts? And if document counts, is this the number of unique documents with a specific code? Please clarify.<br>Total number of EHR records? Please clarify.<br>We have modified the table and caption to make these points clear.<br><br>This section is better-suited under the methods section below. Please update.<br>Moved as requested.<br><br>What is the start date of the data pull? How historical is the cohort?<br>This information has been added.<br><br>Typo? This sentence is a little confusing. Consider revising to "... adequate sample size to make predictive models robust..."<br>Revised as suggested.<br><br>Was this a wildcard text search? Please clarify<br>These are wildcard search terms, clarified in the text as requested.<br><br>You state "high likelihood" but below you note the chart review looked for a positive confirmation of AHP. It sounds like you are in fact confirming AHP through manual chart review.<br>This is correct. Thank you for identifying this confusion. We have revised the text to:<br>To develop a gold standard for the data, a medical student (MN), overseen by clinical experts among the rest of the authors, conducted a chart review to identify patients with a confirmed diagnosis of AHP.<br><br>The remaining 17 records? Please specify.<br>Added clarifying text:<br>For the remaining 17 records, we could not confirm by chart review the diagnosis of AHP. This may be due to the code being attached to the patient based on an encounter to rule out AHP, or a charting error. For these 17 patients no additional information supporting the AHP diagnosis was found in the notes, clinical tests or medication records and the only evidence of AHP was a code in the problem list or

Powered by Editorial Manager® and ProduXion Manager® from Aries Systems Corporation

	encounter diagnosis.<br><br>Results, not methods<br>Results of model building, not methods.<br>The corresponding text has been moved to the results section, and the results section reorganized to incorporate the new text.<br><br>Model? Spelling?<br>Thank you for finding this error. Changed word to “algorithm”.<br><br>What is a source document? The location the field is derived in the EHR? Wouldn't that location depend on the underlying EHR structure? And why is the source document location important?<br>Yes, the source document is dependent upon the underlying structure of the EHR, and of our data warehouse as well. As the EHR itself is a hierarchical patient-oriented database, and our RDW is a relational database extract of that, we have no choice but to treat the records in units corresponding to the structure of the extract. These mappings between the EHR that clinicians use and the data extracts available to investigators is a common situation. The source document types correspond to units of observation common in documenting clinical care electronically. Our feature set provides both the source document and specific data field used in the model in order to provide as much information as possible to anyone trying to repeat our work and perform a similar mapping with their own EHR data. We have tried to make this more clear both in the descriptions, tables, and supplementary data.<br><br>There is no mention of constructing a training dataset in this section until the very end.<br>Thank you for pointing this out. We have added text to clarify how the data was used:<br>The rest of the records were then assumed to be negative for AHP for the purposes of statistical analysis and machine learning. The data set consisted of the positive records plus the presumed negative records. The entire data set was used for statistical analysis and training the machine learning models, the final goal of which was to identify the presumed negative records which are actually likely to be positive.<br><br>Why four patients? What was the rationale for this threshold?<br>Added text:<br>Requiring that included feature have at least four positive case patient records was chosen as a filter to strike a balance between only keeping the most common features, and keeping thousands of rare features requiring manual review that were unlikely be helpful in a generalized model.<br><br>What is the manual review process? Why not simply exclude features for EHR records that also have a corresponding AHP diagnosis, mention or treatment?<br>We could not exclude features as suggested since this criterion would not remove all the biased features and it may remove some associated unbiased features that could be useful.<br>Added: This was done by inspection using clinical domain knowledge.<br><br>How is this process different from the previous "manual review process"? Also, wouldn't the first review (if manual) have identified these same AHP-correlated features?<br>We needed a second pass, which included a clinical porphyria expert, to ensure that we did not miss any features that were biased by clinical pre-existing knowledge of a diagnosis of porphyria for the patient.<br>Added text:<br>This second pass incorporated a higher level of clinical expertise than the first pass. It was performed after filtering by SVM weight in order to reduce the screening load on our clinical expert.<br><br>I would expect the results section to begin with this number, highlighting the total number of patients in the entire dataset, then the final number of patients used for subsequent analyses.<br>Moved this text to the beginning of the results section.<br><br>General comment on all tables- please update the tables so they share the same

Powered by Editorial Manager® and ProduXion Manager® from Aries Systems Corporation

	format throughout the paper (e.g. font, font size, bold use, number formats).<br>We have reformatted the tables to use a consistent style.<br><br>Total number of EHR records? Please clarify.<br>Total number of EHR documents and patient records added to caption for Table 2.<br><br>Unique patients, or unique records/document counts? And if document counts, is this the number of unique documents with a specific code? Please clarify.<br>Clarified in table caption and column headings.<br><br>Please spell out the document types. The current list appears to be table names from the database itself. For example, "current_medications" should be renamed "Concomitant Medications" or "Poly-Pharmacy". "demographics" should be "Patient Demographics". I also recommend providing a brief description of these fields, as some readers may not be as familiar with traditional EHR domains.<br>I recommend including standard deviation with any results presenting Mean.<br>Finally, be sure to format the table numbers (some rows appear to have comma delimiters, others do not).<br><br>Table 3 document type names changed to correspond with the document types in Table 1. Reformatted numbers to not use commas.<br>Table has been reformatted to be consistent and use full document names. Data dictionary definitions of the document types has been added to Table 1 to describe what is in these documents. Mean has been removed as table is too wide with the additions and larger font. Median and max remain and are sufficiently informative for this purpose.<br><br>Please provide either a data dictionary with descriptions for each feature, or update this table with descriptions of each feature. The current format requires the reader to assume what each feature represents based on the feature dataset name, but formal descriptions would provide more explicit clarity for the reader.<br><br>Table has been reformatted and extended to include data descriptions.
**Additional Information:**	
**Question**	**Response**
**Financial Disclosure**<br><br>Enter a financial disclosure statement that describes the sources of funding for the work included in this submission. Review the submission guidelines for detailed requirements. View published research articles from *PLOS ONE* for specific examples.<br><br>This statement is required for submission and **will appear in the published article** if the submission is accepted. Please make sure it is accurate.	AC, BH, SC, and MN received support for this work from Alnylam Pharmaceuticals, Inc., Cambridge, MA.<br><br>SM, JK, JA and AW are/were employees of Alnylam Pharmaceuticals, Inc., Cambridge, MA during the time of this research.<br><br>This work was funded and the associated editorial support was provided by Alnylam Pharmaceuticals, Inc., Cambridge, MA.<br>Grant number 4510005336<br>https://www.alnylam.com/<br><br>Alnylam participated in algorithm design and preparation of the manuscript. They had no role in the evaluation or EHR data collection and analysis, nor did they have any access to the individual patient electronic health record data used in this research.

**Unfunded studies**<br>Enter: *The author(s) received no specific funding for this work.*<br><br>**Funded studies**<br>Enter a statement with the following details:<br>• Initials of the authors who received each award<br>• Grant numbers awarded to each author<br>• The full name of each funder<br>• URL of each funder website<br>• Did the sponsors or funders play any role in the study design, data collection and analysis, decision to publish, or preparation of the manuscript?<br>• **NO** - Include this sentence at the end of your statement: *The funders had no role in study design, data collection and analysis, decision to publish, or preparation of the manuscript.*<br>• **YES** - Specify the role(s) played.<br><br>* typeset	
**Competing Interests**<br><br>Use the instructions below to enter a competing interest statement for this submission. On behalf of all authors, disclose any competing interests that could be perceived to bias this work—acknowledging all financial support and any other relevant financial or non-financial competing interests.<br><br>This statement **will appear in the published article** if the submission is accepted. Please make sure it is accurate. View published research articles from *PLOS ONE* for specific examples.	I have read the journal's policy and the authors of this manuscript have the following competing interests:<br><br>GIVLAARI is a product of Alnylam. GIVLAARI is a prescription medicine used to treat acute hepatic porphyria (AHP) in adults.

Powered by Editorial Manager® and ProduXion Manager® from Aries Systems Corporation

**NO authors have competing interests**<br><br>Enter: *The authors have declared that no competing interests exist.*<br><br>**Authors with competing interests**<br><br>Enter competing interest details beginning with this statement:<br><br>*I have read the journal's policy and the authors of this manuscript have the following competing interests: [insert competing interests here]*<br><br>* typeset	
**Ethics Statement**<br><br>Enter an ethics statement for this submission. This statement is required if the study involved:<br><br>• Human participants<br>• Human specimens or tissue<br>• Vertebrate animals or cephalopods<br>• Vertebrate embryos or tissues<br>• Field research<br><br>Write "N/A" if the submission does not require an ethics statement.<br><br>General guidance is provided below. Consult the submission guidelines for detailed instructions. **Make sure that all information entered here is included in the Methods section of the manuscript.**	This study protocol was approved by the OHSU Institutional Review Board (IRB00011159).

**Format for specific study types**<br><br>**Human Subject Research (involving human participants and/or tissue)**<br>• Give the name of the institutional review board or ethics committee that approved the study<br>• Include the approval number and/or a statement indicating approval of this research<br>• Indicate the form of consent obtained (written/oral) or the reason that consent was not obtained (e.g. the data were analyzed anonymously)<br><br>**Animal Research (involving vertebrate animals, embryos or tissues)**<br>• Provide the name of the Institutional Animal Care and Use Committee (IACUC) or other relevant ethics board that reviewed the study protocol, and indicate whether they approved this research or granted a formal waiver of ethical approval<br>• Include an approval number if one was obtained<br>• If the study involved *non-human primates*, add *additional details* about animal welfare and steps taken to ameliorate suffering<br>• If anesthesia, euthanasia, or any kind of animal sacrifice is part of the study, include briefly which substances and/or methods were applied<br><br>**Field Research**<br><br>Include the following details if this study involves the collection of plant, animal, or other materials from a natural setting:<br>• Field permit number<br>• Name of the institution or relevant body that granted permission	
**Data Availability**<br><br>Authors are required to make all data underlying the findings described fully available, without restriction, and from the time of publication. PLOS allows rare exceptions to address legal and ethical concerns. See the PLOS Data Policy and FAQ for detailed information.	No - some restrictions will apply

Powered by Editorial Manager® and ProduXion Manager® from Aries Systems Corporation

A Data Availability Statement describing where the data can be found is required at submission. Your answers to this question constitute the Data Availability Statement and **will be published in the article**, if accepted.<br><br>**Important:** Stating 'data available on request from the author' is not sufficient. If your data are only available upon request, select 'No' for the first question and explain your exceptional situation in the text box.<br><br>Do the authors confirm that all data underlying the findings described in their manuscript are fully available without restriction?	
**Describe where the data may be found in full sentences. If you are copying our sample text, replace any instances of XXX with the appropriate details.**<br><br>• If the data are **held or will be held in a public repository**, include URLs, accession numbers or DOIs. If this information will only be available after acceptance, indicate this by ticking the box below. For example: *All XXX files are available from the XXX database (accession number(s) XXX, XXX.*).<br>• If the data are all contained **within the manuscript and/or Supporting Information files**, enter the following: *All relevant data are within the manuscript and its Supporting Information files.*<br>• If neither of these applies but you are able to provide **details of access elsewhere**, with or without limitations, please do so. For example:<br><br>*Data cannot be shared publicly because of [XXX]. Data are available from the XXX Institutional Data Access / Ethics Committee (contact via XXX) for researchers who meet the criteria for access to confidential data.*<br><br>*The data underlying the results presented in the study are available from (include the name of the third party*	The source data used for this project is electronic health record (EHR) data, and contains protected health information (PHI) for patients under care at Oregon Health & Science University (OHSU). The OHSU Institutional Review Board (IRB) does not allow release of this data to the public, and doing so would violate US HIPAA laws. The OHSU IRB can be contacted at: irb@ohsu.edu. Questions about data requests may be sent to this address.<br><br>We are including full details of the machine learning model, training methods, and final features. Other investigators experienced in the field should be able to reproduce our methods on their own data to validate the results presented in this manuscript.

and contact information or URL).<br>• This text is appropriate if the data are owned by a third party and authors do not have permission to share the data.<br><br>* typeset	
Additional data availability information:	Tick here if your circumstances are not covered by the questions above and you need the journal's help to make your data available.

Powered by Editorial Manager® and ProduXion Manager® from Aries Systems Corporation

Revised Manuscript
Click here to access/download;Manuscript;AHP_ML_PLOS_MEDICINE_revis

Detecting Rare Diseases in Electronic Health Records Using Machine Learning and Knowledge Engineering: Case Study of Acute Hepatic Porphyria

Aaron Cohen, MD, MS [1*]
Steven Chamberlin, ND [1]
Thomas Deloughery, MD [1]
Michelle Nguyen, BS [1]
Steven Bedrick, PhD [1]
Stephen Meninger, PharmD [2]
John J. Ko, PharmD, MS [2]
Jigar Amin, PharmD [2]
Alex Wei, PharmD [2]
William Hersh, MD [1]

[1]Department of Medical Informatics & Clinical Epidemiology, School of Medicine, Oregon Health & Science University, Portland, OR USA.

[2]Alnylam Pharmaceuticals, Cambridge, MA, USA.

* Corresponding Author:
Aaron M. Cohen, MD MS
Professor
Department of Medical Informatics & Clinical Epidemiology
School of Medicine
Oregon Health & Science University
Portland, Oregon USA 97239
Email: cohenaa@ohsu.edu

Abstract

Background

With the growing adoption of the electronic health record (EHR) worldwide over the last decade, new opportunities exist for leveraging EHR data for detection of rare diseases. Rare diseases are often not diagnosed or delayed in diagnosis by clinicians who encounter them infrequently. One such rare disease that may be amenable to EHR-based detection is acute hepatic porphyria (AHP). AHP consists of a family of rare, metabolic diseases characterized by potentially life-threatening acute attacks and, for some patients, chronic debilitating symptoms that negatively impact daily functioning and quality of life. The goal of this study was to apply machine learning and knowledge engineering to a large extract of EHR data to determine whether they could be effective in identifying patients not previously tested for AHP who should receive a proper diagnostic workup for AHP.

Methods and Findings

We used an extract of the complete EHR data of 200,000 patients from an academic medical center for up to 10 years longitudinally and enriched it with records from an additional 5,571 patients from the center containing any mention of porphyria in notes, laboratory tests, diagnosis codes, and other parts of the record. After manually reviewing the records of all 47 unique patients with the ICD-10-CM code E80.21 (Acute intermittent [hepatic] porphyria), we identified 30 patients who were positive cases for our machine learning models, with the rest of the patients used as negative cases. We parsed the record into features, which were scored by frequency of appearance and labeled by the EHR source document. We then carried out a univariate feature analysis, manually choosing features not directly tied to provider attributes or suspicion of the patient having AHP. We next trained on the full dataset, with the best cross-validation performance coming from support vector machine (SVM) algorithm using a radial basis function (RBF) kernel. The trained model was applied back to the full data set and patients were ranked by margin distance. The top 100 ranked negative cases were manually reviewed for symptom complexes similar to AHP, finding four patients where AHP diagnostic testing was likely indicated and 18 patients where AHP diagnostic testing was possibly indicated. From the top 100 ranked cases of patients with mention of porphyria in their record, we identified four patients for whom AHP diagnostic testing was possibly indicated and had not been previously performed. Based solely on the reported prevalence of AHP, we would have expected only 0.002 cases out of the 200 patients manually reviewed.

Conclusions

The application of machine learning and knowledge engineering to EHR data may facilitate the diagnosis of rare diseases such as AHP. The only manual modifications to this work were the removal of disease-specific or medical center specific features that might undermine our ability to find new cases. Further work will recommend clinical investigation to identified patients' clinicians, evaluate more patients, assess additional feature selection and machine learning algorithms, and apply this methodology to other rare diseases.

Introduction

The growing adoption of the electronic health record (EHR) worldwide has created new opportunities for leveraging EHR data for other, so called *secondary* purposes, such as clinical and translational research, quality measurement and improvement, patient cohort identification and more (1). One emerging use case for leveraging of EHR data is to detect undiagnosed rare diseases. Although there is no absolute definition of a rare disease, the US Rare Diseases Act of 2002 defines rare diseases as those that occur in fewer than 200,000 patients worldwide (2), and the National Organization for Rare Disorders (NORD, https://rarediseases.org/) registry lists more than 1,200 diseases. Others have noted that the true number of rare diseases is unknown, and have called for more research to define them (3).

Rare diseases can be difficult to diagnose because their infrequent occurrence may result in primary care physicians not considering them in diagnostic workups (4). They also often have general presentations with diffuse symptoms, as well as genetic components which may require specialized testing. This lack of timely diagnosis may lead to both physical and emotional suffering as patients remain undiagnosed for prolonged periods. Additionally, a lack of accurate diagnoses increases economic burden to healthcare systems as patients continue to receive inadequate and/or inappropriate treatment. Some informatics researchers have used EHR data to detect rare diseases, such as cardiac amyloidosis (5), lipodystrophy (6), and a large collection of different diseases (7, 8).

One rare disease that may be amenable to EHR-based detection is acute hepatic porphyria (AHP). AHP is a subset of porphyria that refers to a family of rare, metabolic diseases characterized by potentially life-threatening acute attacks and, for some patients, chronic debilitating symptoms that negatively impact daily functioning and quality of life (9-13). During attacks, patients typically present with multiple signs and symptoms due to dysfunction across the autonomic, central, and peripheral nervous systems. The prevalence of diagnosed symptomatic AHP patients is ~1 per 100,000 (14). Due to the nonspecific symptoms and the rare nature of the disease, AHP is often initially overlooked or misdiagnosed. A U.S. study demonstrated that diagnosis of AHP is delayed on average by up to 15 years (15).

AHP is predominantly caused by a genetic mutation leading to a partial deficiency in the activity of one of the eight enzymes responsible for heme synthesis (12). These defects predispose patients to the accumulation of neurotoxic heme intermediates aminolevulinic acid (ALA) and porphobilinogen (PBG) when the rate limiting enzyme of the heme synthesis pathway, aminolevulinic acid synthase 1 (ALAS1), is induced (10, 16). Gene mutations causing the disease are mostly autosomal dominant, however the disease has low penetrance (~1%) and many specific mutations have not been identified (17). Furthermore, families carrying the gene may have few or only one affected member. Therefore, family history can be a poor diagnostic tool for this disease. The preferred diagnostic procedure for AHP is biochemical testing of random/spot urine for ALA, PBG, and porphyrins (18, 19).

Historically, treatment of AHP has predominantly focused on avoidance of attack triggers, management of pain and other chronic symptoms, and treatment of acute attacks through the use of Panhematin® (hemin for injection) (20). Panhematin was FDA approved in 1983 for the

amelioration of recurrent attacks of acute intermittent porphyria (AIP) temporally related to the menstrual cycle in susceptible women after initial carbohydrate therapy is known or suspected to be inadequate.

Recently, a new drug Givlaari® (givosiran), for subcutaneous injection has been approved by the FDA for the treatment of adults with AHP (21). Givosiran is a double-stranded small interfering RNA (siRNA) molecule that reduces induced levels of the protein ALAS1. A Phase 1 trial has been published (22) and a Phase 3 randomized control trial has shown this therapy to be effective in reducing the occurrence of acute attacks and impacting other manifestations of the disease (21).

Materials and Methods

This study protocol was approved by the OHSU Institutional Review Board (IRB00011159).

Dataset

Oregon Health & Science University (OHSU) is the only academic medical center in Oregon and is thus a referral center for rare diseases like AHP. The OHSU Research Data Warehouse (RDW) is a research data "honest broker" service that provides EHR data to researchers, with appropriate IRB approval. The investigators have an ongoing institutional review board (IRB) approval to use an extract from the Oregon Health & Science University (OHSU) EHR research data warehouse (RDW) for a series of patient cohort identification projects. For this research, the patient cohort to identify was defined as those patients who have a documented clinical history of AHP, or a clinical history indicating that AHP diagnostic testing may be appropriate. The goal of this study was to apply machine learning and knowledge engineering to a large extract of EHR data to determine whether the combined approach could be effective in identifying patients not previously tested for AHP who should receive a proper diagnostic workup for AHP.

A large dataset of approximately 200,000 patient records was requested from the RDW, complete as of the data pull date in March 2019, including over 30 million text notes plus other document types. The data set goes back to the start of OHSU using the Epic EHR system in January, 2009. These records consist of all patients who had more than one primary care health care visit at our institution. Each patient record was represented as a collection of documents of types given in **Table 1**. Patient records could include zero or more documents of each type.

To insure an adequate sample size to make predictive models robust, we enriched the data set for possible AHP by adding records from an additional 5,571 patients who met one or more of the following case-insensitive criteria (see **Table 2**):

- Diagnosis including the wildcard search term "porph*" in the diagnosis name
- Medication including the wildcard search term "hemin*" in the medication name
- Procedure including the wildcard search term "porph*" in the procedure name
- Clinical or result note including the wildcard search term "porph*" in the note text

To develop a gold standard for the data, a medical student (MN), overseen by clinical experts among the rest of the authors, conducted a chart review to identify patients with a confirmed diagnosis of AHP. We manually reviewed all the patients with the ICD-10-CM code E80.21

(Acute intermittent [hepatic] porphyria) in their record, looking for positive confirmation of AHP either through a lab test or a specific comment in a progress note. This process yielded 30 positive cases from the 47 coded for E80.21. As OHSU is the only academic medical center in Oregon and is thus a referral center for rare diseases like AHP, this may explain why the number of identified AHP patients in our database was higher than that which would be expected based on the global prevalence of AHP. For the remaining 17 records, we could not confirm by chart review the diagnosis of AHP. This may be due to the code being attached to the patient based on an encounter to rule out AHP, inaccurate past medical history data, or a charting error. For these 17 patients no additional information supporting the AHP diagnosis was found in the notes, clinical tests or medication records and the only evidence of AHP was an ICD-10-CM code at one place in the medical record.

The rest of the records were then assumed to be negative for AHP for the purposes of statistical analysis and machine learning. The data set consisted of the positive records plus the presumed negative records. The entire data set was used for statistical analysis and training the machine learning models, the final goal of which was to identify the presumed negative records which are actually likely to be positive.

We then deconstructed each patient record into a number of features to be used for machine learning. Structured data fields were encoded directly with the entire field content used as the feature. Free-text fields were parsed into unigrams and bigrams.

All features were labeled with their source document fields. This enabled, for example, diagnosis names in ICD-10-CM code fields in the problem list to be distinguished from the same text appearing in free text notes. Feature values were encoded as the number of occurrences in the entire record for the patient. A summary of the types and counts of documents in the data set is shown in **Table 3**.

Feature Selection and Machine Learning Methods

Features to be included in the machine learning model were selected by performing univariate logistic regression analysis of the entire feature set, using the confirmed AHP patients as positive samples and the rest of the data set as negative samples. For each document type, the 100 top features were chosen, ranked by odds ratio, having a p-value < 0.01 and occurring in at least 4 positive case patient records. This statistical criteria was used to establish which data elements had a significant relationship between the outcome variable, which was the presence, or not, of a confirmed diagnosis of AHP. Requiring that included features have at least four positive case patient records was chosen as a filter to strike a balance between only keeping the most common features, and keeping thousands of rare features requiring manual review that were unlikely be helpful in a generalized model.

From these several hundred features, a manual review process was performed to ensure that none of these features were directly connected to a diagnosis of AHP, mention of AHP in the record, or treatment of AHP. This was done by inspection. This process eliminated all text features mentioning any bigram of “acute hepatic porphyria,” medications such as hematin, and laboratory codes that in the OHSU system represented tests specifically for the diagnosis of porphyria.

The remaining features were then evaluated by using them in a machine learning model and scoring the model using 5 repetitions of 2-fold cross-validation. Several SVM kernel functions were tested including linear, polynomial degree 2, and the radial basis function (RBF), random forests, Adaboost, J48, and several topologies of Neural Network. Two normalization encoding methods were tried as well, binary, linear and log normalizing feature occurance counts beween 0.0 and 1.0.

After algorithm selection, a second round of feature screening was performed. Any features with non-zero algorithm weights were removed if any direct connection to AHP could be established. This was performed by close scrutiny and discussion with our clinical expert for each feature. This second pass incorporated a higher level of clinical expertise than the first pass. It was performed after filtering by machine learning weights in order to reduce the screening load on our clinical expert.

Machine Learning for AHP Prediction and Evaluation Methodology

A final trained model using the features selected was created by training the selected algorithm with chosen parameter settings on the entire data set. This model was then applied back to the entire data set in order to create an AHP prediction score for each patient. The classifier margin distance was taken as the prediction score.

The patient prediction scores were then analyzed. To keep the manual chart review process manageable, we could not review every patient. We decided to review the top scoring 100 cases manually from each of two subsets of the general population.

The first reviewed subset of 100 patients were those with no mention of porphyria in their chart, no related ICD-9-CM or ICD-10-CM codes, and no porphyria specific lab test. We selected the top scoring 100 patients that met these criteria. This represents the most important target population for our project – patients with persistent symptoms that have not had AHP considered and tested to rule it in or out as a diagnosis. Manual review of these cases is intended to demonstrate the potential of our proposed approach to identify potential cases of AHP that would benefit from diagnostic testing and follow up.

The second reviewed subset of 100 patients were those with a mention of porphyria in the text notes in their chart, but no related ICD-9-CM or ICD-10-CM diagnosis codes, and no porphyria-specific lab test. These are patients where porphyria may have been considered by the clinician, or may have been tested at another health care facility with unavailable records, or may have been a work up in progress. Manual review of these cases was intended to discern the clinical face validity of the algorithmic predictions, that is, the high scoring patients in this group score high because the algorithm is paying attention to some of the same non-AHP-specific clinical symptoms and other variables as the clinician. While the manual review of these patients was primarily intended for gaining insight into how the algorithm was scoring patients with porphyria mentioned in the charts, based on the manual review some patients who may benefit from diagnostic testing could be found.

A clinically trained reviewer assessed the patients' records in these two non-overlapping subsets for symptom patterns consistent with acute hepatic porphyria (AHP). The reviewer was blinded to the model features. Clinical notes were searched for the 'classic triad' of AHP symptoms: abdominal pain, central nervous system abnormalities, and peripheral neuropathy (23). In

addition, any report of pain was assessed, and searches were also conducted for the highest incident AHP symptoms: abdominal pain, vomiting, constipation, muscle weakness, psychiatric symptoms, limb, head, neck, or chest pain, hypertension, tachycardia, convulsion, sensory loss, fever, respiratory paralysis, diarrhea (23). All major comorbidities were also reviewed and documented, as well as alternative diagnoses to explain AHP symptom profiles.

The 100 patients with no mention of porphyria in their EHR record were classified into one of three categories: *AHP diagnostic testing likely indicated, AHP diagnostic testing possibly indicated,* and *AHP diagnostic testing unlikely indicated.* To be classified as *likely*, symptoms had to be present in all three categories of the 'classic triad', without a cause identified in the EHR, and with a substantial history of symptoms. To be classified as *possibly*, symptoms had to be present in at least one of the three categories, without a cause documented and with a substantial history. Patients were classified as *unlikely* if their symptoms could be explained by another diagnosis, or if they did not have a strong AHP symptom profile.

The 100 patients who did have a mention of porphyria in their clinical notes were classified into one of five categories of AHP status based on chart review and details in the clinical notes: *AHP already suspected, AHP already suspected but ruled out*, *diagnostic testing likely indicated but AHP not suspected*, *unlikely AHP*, and *AHP diagnosis mentioned in notes*. A patient was classified as *AHP already suspected* if there was any level of AHP suspicion mentioned in their clinical notes, without a formal diagnosis or lab test. *AHP already suspected but ruled out* was assigned if there was a suspicion of AHP in the note, but had been ruled out, usually by negative lab tests. These lab tests were only documented in the note, since we excluded patients from this subset who had lab tests in the laboratory data itself. *Diagnostic testing likely indicated but AHP not suspected* was assigned if there were symptoms present in at least one of the three triad categories, without a cause, but no suspicion of AHP mentioned in the notes. For these patients the clinical notes contained the string 'porph' but presence of 'porph' in the clinical note was not related to suspicion of AHP. *Unlikely AHP* was assigned if AHP type symptoms could be explained by another diagnosis, or there was not a strong AHP symptom profile. Finally, patients were assigned to *AHP diagnosis* if there was any mention of an existing AHP diagnosis in the notes, even patient reported. The reasons for the presence of the string 'porph' in the clinical note for the second set of 100 patients was also reviewed and documented. Patient's categorized as *AHP already suspected* and *Diagnostic testing likely indicated but AHP not suspected* would benefit from AHP testing as they displayed suspicion of AHP or symptom complexes associated with AHP but have yet received a full diagnostic work-up.

Results

Final selected features and machine learning cross-validation

Figure 1 shows a flowchart of the overall patient record filtering and manual review process. The process starts with 204,413 patient records, and using a combination of machine learning and structured data filtering described above, identifies 200 patients that were manually reviewed. 100 of those patients were identified as not having any mention of porphyria in the medical record and potentially could benefit from AHP diagnostic testing. The other 100 of those patients did have mention of porphyria in their medical record, but no diagnostic code for porphyria. These records were reviewed to determine the reason for the mention of porphyria and evaluate whether these reasons were consistent with the goal of the machine learning to identify patients with symptoms and other clinical features consistent with a possible porphyria diagnosis.

Several hundred features made it through the statistical testing and occurrence frequency filter. From these several hundred features, the manual review process reduced the set to approximately 200 features. These features were then evaluated by using them in a machine learning model and scoring the model using 5 repetitions of 2-fold cross-validation. These experiments found that an SVM with the radial basis function (RBF) kernel scored best for the ranking metrics AUC and average precision. The other machine learning methods explored failed to perform as well as the RBF SVM. It was also determined that feature values were best encoded using log normalization, transforming feature occurrence counts into values between 0.0 and 1.0. Binary encoding, as well as linear normalization, failed to perform as well. We used the SVMLight implementation of the RBF kernel. Experimentation with cross-validation showed gamma = 0.04 to be optimal.

After algorithm selection and tuning, the second round of feature screening removed a few features that the SVM model assigned non-zero weights which were thought to be directly connected to the pre-established diagnosis of AHP by the clinical expert. For example, based on case series evidence, clinical hematology AHP specialists sometimes use cimetidine to treat AHP symptoms, as it is known to block a portion of the heme synthesis pathway as a side effect (24). We found that cimetidine was a highly weighted feature in our initial models (due to its use by a specialist [TD] at OHSU based on case report data (24)) that had to be removed as it is given in response to AHP rather than being predictive. This process resulted in 141 total features being included in the final model.

The 141 features included in the final model are shown in **Table S-1**. Final feature set cross-validation performance on the entire training set is shown in **Table 4**.

Application of machine learning to the full data set

The final machine learning model with the 141 features was trained on the entire data set, and this model was then applied back to the entire data set in order to provide a margin distance score for every patient.

The patient prediction scores were then analyzed. In particular, the range of scores obtained for the 30 confirmed positive training cases were compared to the rest of the patients in the data set. About 22,000 patients in the general population had scores that overlapped with those of the 30 positive patients. While this was only 10% of the patient records, it was more than could be manually reviewed.

We reviewed the top scoring 100 cases manually from each of two subsets of the general population. Out of the 100 patient charts we reviewed with no mention of porphyria, four were identified as likely to *AHP diagnostic testing likely indicated*, all without mention of porphyria in their medical record or documentation of a urine PBG test. The first patient was a male with six years of unexplained intermittent abdominal pain with nausea, vomiting, and diarrhea. His other conditions included complex regional pain syndrome, peripheral neuropathy, cardiac arrhythmias, panic attacks, and depression. The next patient was a female whose abdominal pain was described as ‘a long standing symptom with extensive negative evaluation’. Also listed in her profile were neuralgias, hereditary small fiber neuropathy, movement disorder, fibromyalgia, migraines, palpitations, and somatization disorder. The third patient was a woman with multiple emergency department admissions for severe abdominal pain. She also had severe suicidality with a permanent tracheostomy due to a hanging attempt, borderline personality disorder,

tachycardia, anxiety, saddle anesthesia, insomnia, and severe somatization disorder including a comment in her note advising not to admit the patient for only vague complaints. The fourth patient was a female with a history of abdominal pain comments in the notes describing that the etiology had not been identified for her complex symptomology which included headaches, abdominal pain, paresthesias and palpitations.

Overall, about a quarter of the 100 patients in the group without mention of porphyria had symptom profiles that were consistent with undiagnosed AHP and AHP diagnostic testing would either be likely or possibly indicated (**Table 5**). In this group there was no sign or suspicion of AHP by the clinician in the record. This is a much higher concentration of possible AHP patients than would be expected by chance based on the known prevlance of AHP.

Alternate explanations for characteristic AHP symptom profiles were diverse in the patient group without any mention of porphyria (**Table 6).** Cancers seen in this group included breast, uterine, pancreatic, cervical, leukemia and adrenal carcinoma. Other common comorbidities and conditions seen in this group included: fibromyalgia, irritable bowel syndrome, chronic fatigue, obesity, hypertension, obstructive sleep apnea, and chronic obstructive pulmonary disease. In contrast, alternate symptom profiles in the group with mention of porphyria in the notes were dominated by liver pathologies, mostly hepatocellular carcinoma.

Patients in the group *without* mention of porphyria in the medical record generally had much longer and more complicated histories compared to the other group, with 86 out of 100 having encounters spread over four years or longer. The patients *with* porphyria mentioned in the clinical notes tended to have shorter, and less complex histories (only 39 out of 100 had over 4 years of encounters), more focused on a single medical issue or set of symptoms, which may have been due to their being referral to our academic medical center from other health care sites.

There were small differences in age summary statistics between the two groups (**Table 7**), but notably more pediatric patients in the reviewed group with mention of porphyria found in clinical notes than those without (10 patients vs 1 patient). There were significantly more male patients found in this group too, compared to the group with no mention of porphyria (**Table 8**). Associated conditions for these 44 male patients were dominated by only a few diagnoses/symptom patterns: liver disease (N=18), suspicion of porphyria (N=11), or actinic keratosis (N=3). In contrast, no single condition dominated the male disease distribution in the patient group without mention of porphyria in the notes.

About a third of patients in the group *with* mention of porphyria in the clinical notes had some level of suspicion and work-up for AHP documented. We also identified four patients in this group that we thought had possibly undiagnosed AHP, without suspicion documented in the notes. We labeled these patients as *Diagnostic testing likely indicated but AHP not suspected.* Three of these patients had 'porphyria' in their clinical note listed as a standard precaution for several different medications (hydrochloroquinone, ferrous sulfate), which they were taking. In fact, about two thirds of the patients with 'porphyria' in the clinic notes had other reasons, besides suspicion of AHP, for the presence of this word (**Table 9**). A large number of these patients were candidates for liver transplantation. Standard clinical documentation for evaluation for this procedure included a list of possible causes of liver failure, including protoporphyria.

Porphyria was also mentioned as a precaution for certain medications or treatments given to some patients in this group, which included hydroxycholorquinone ferrous sulfate, therapeutic abortion, and UV light therapy for actinic keratosis.

Discussion

This work identified four likely and 18 possible patients who had no mention of porphyria in their charts for whom AHP diagnostic testing could be indicated. In addition, four patients who had mention of porphyria in their charts not related to a diagnostic evaluation of the disease were also found likely to have AHP diagnostic testing indicated. This number of patients with indications for AHP diagnostic testing and possibly to-be confirmed diagnosis vastly exceeds that due to chance and surpassed our expectations. It will require clinical follow-up to determine whether these patients' symptoms are truly due to AHP or not, but the manual record review clearly demonstrates that our methodology has found patients for whom a spot urine porphobilinogen test is indicated.

Another benefit of identifying such patients is to inform local specialists of the presence of patients with rare diseases in which they have expertise. An institution-wide search for confirmed AHP patients through our targeted ICD-10-CM code search plus manual chart review identified 30 confirmed AHP patients. A majority of these patients were previously unknown to the porphyria specialist (TD) at OHSU. Identifying rare disease patients through large-scale data review in this manner can help connect them with the appropriate specialist to ensure optimal care.

Our results strongly suggest that leveraging of EHR data coupled with machine learning can be an effective method of identifying patients who should receive a diagnostic biochemical test to screen for AHP. Our automated model was able to identify patients with compelling constellations of symptoms who had not be previously worked up for porphyria. It was also able to identify patients for whom porphyria had been considered without direct access to porphyria-related data elements such as hemin treatment, lab tests specific to AHP, or mention of AHP diagnosis in clinical notes.

This is especially interesting in the light that the overall cross-validation scores of the model on the data set using the known 30 AHP cases as the positive set and the rest of the data as negative training samples was not very high, with cross-validation yielding an average AUC = 0.775. This is certainly a low performance figure compared to other current machine learning tasks such as publication type identification (25), or facial image recognition (26). However, these other tasks are very different from this one due to the extremely rare nature of the positive AIP cases in both the training data as well as in the actual patient population. In most machine learning research, a data set is considered skewed or imbalanced if the number of positive cases is much less than 50%. A recent systematic review on imbalanced data classification cites articles investigating negative to positive case ratios of 100 to 1 as "highly imbalanced" (27, 28). For problems such as rare diseases, the imbalance ratio can be nearly 10,000 to 1, as it is here. Lifting the predictive power to perhaps 22 in 100 manually reviewed cases is a potentially transformative level of performance.

The strongest positive predictors in the model included unexplained abdominal pain, pelvic and perineal pain, nausea and vomiting, and a number of pain and nausea medications. Frequent urinalysis was also a strong positive predictive feature, this is likely due to being associated with frequent ER visits and hospitalizations. The model relied on encoding the frequency of episodes, and not just binary presence of absence of symptoms. Indirectly, in the model this represented recurrent, undiagnosed problems consistent with AHP.

As these methods are general, and not specific to AHP, they should be applicable to other rare disorders that have a constellation of recurrent symptoms as indicating features. There are likely ways to improve the machine learning approach, including the use of more advanced features that represent time, duration, and intervals, explicit coding of symptom separation and overlap, and more sophisticated machine learning algorithms specifically tailored to situations where the positive case is extremely rare. Investigation into machine learning algorithms for highly skewed data such as these is an active area of research (29).

Conclusion

The combination of large data sets, machine learning techniques, and clinical knowledge engineering can be a powerful tool to identify patients with undiagnosed rare diseases. The use case of AHP presented here revealed four undiagnosed patients thought likely to have AHP, as well as 18 others who would likely benefit from testing. This level of precision in identifying potential cases of AHP from EHR data is much higher than would be expected by the prevalence of the disease.

Analyzing the EHR with advanced techniques such as demonstrated here points to the potential of the future of digital medicine on a population scale. Advanced approaches enabled by the wide deployment of the EHR can now be used to improve medicine and medical care in areas that have been underserved or inaccessible. Health care can be made more proactive, not simply in terms of common conditions and age or gender related screening, but for rarer conditions as well.

We plan to continue this work in several directions. First, an IRB-approved clinical validation study is being implemented. In this study, we will contact the primary care clinicians (PCP) of the patients where AHP diagnostic testing was found to be *likely* or *possibly* indicated. We will inform them that an algorithm based on EHR data has determined that their patient might have AHP and could benefit from a spot urine porphobilinogen, which is an is inexpensive, non-invasive and easy to perform diagnostic test. With the agreement of the PCP, we will then contact patients and offer them the test. Expert clinical consultation will be made available to the PCP for any questions they have. We will collect data on the interactions with the PCPs, the number of spot urine porphobilinogen tests administered, as well as the test results. In this manner, we will be able to study the clinical impact of our rare disease identification approach.

Second, we will continue to refine our methods. Other machine learning algorithms, such as random forests and deep learning, may have advantages for AHP and other rare diseases. Other methods of encoding the EHR data that incorporate embeddings and temporal representations,

have been shown to demonstrate leading-edge results in other fields, such as computer vision, machine translation, and speech recognition, and may assist with rare diseases.

Finally, we will extend this methodology to other rare diseases that are difficult to diagnose, focusing on those for which effective treatments are becoming available. If the timeline for diagnosing rate conditions can be substantially reduced, there is great potential to impact patient health in a very significant manner.

Acknowledgements and Funding

This work was funded and the associated editorial support was provided by Alnylam Pharmaceuticals, Inc., Cambridge, MA.

Declaration of Interest

Stephen Meninger, John J. Ko, and Jigar Amin, are employees of Alnylam, and Alex Wei was an employee of Alnylam during his contribution to the manuscript.

Table 1. Electronic Health Record (EHR) document types used in this research.

EHR Document Record Type	Description of Document
Administered Medications	Medications given to patient during a hiospital stay or ambulatory encounter.
Current Medications	The concomittent medications a patient is taking, as documented by providers during encounters.
Demographics	Patient demographic information
Encounter Diagnosis	The diagnoses and diagnostic codes assigned to a patient ambulatory encounter.
Hospital Encounters	Patient-level hospital admission information including times and billing codes.
Lab Results	Results of ordered lab tests including order time.
Medications Ordered	Medications ordered by for patients by clinicians during an encounter.
Microbiology Results	Results of microbiology lab tests in text form.
Notes	All types of clinical text including progress notes and discharge summaries.
Problem List	The concomittent list of active medical issues for a patient, as documented by providers during encounters.
Procedures Ordered	Procedures ordered by clinicians for patients during an encounter.
Lab Result Comments	Non-numerical, text portion, if any for results of lab tests.
Surgeries	Description of surgeries performed on patient at hospital in both text and coded forms.
Vitals	Documentation of vital values such as heartrate, blood pressure, weight, and temperature.

Table 2. Electronic Health Record (EHR) total document and unique patients counts of porphyria codes and mentioned in text notes or label tests. Counts shown here are out of a total of 347,709,284 individual EHR documents and 204, 413 total unique patient records.

**Code**	**Total Documents**	**Total Patients**
ICD9 277.1	3879	308
E80.0 Hereditary erythropoietic porphyria	472	37
E80.1 Porphyria cutanea tarda	783	77
E80.20 Unspecified porphyria	2010	247
E80.21 Acute intermittent (hepatic) porphyria	1016	47
E80.29 Other porphyria	109	24
E80.4 Gilbert syndrome	3197	366
E80.6 Other disorders of bilirubin metabolism	9502	2308
E80.7 Disorder of bilirubin metabolism, unspecified	75	58
Patients with porphyria mentioned in a lab test:	359	175
Searching field NOTE_TEXT for term porphyria:	14353	3012

Table 3. Summary of document types and counts used in the EHR data set for this research.

Document Type	Patients	Encounters	Records	Median	Max
Current Medications	187724	N/A	99602443	89	57406
Demographics	204413	N/A	204413	1	1
Encounter Attributes	204412	19589057	19589057	43	3335
Encounter Diagnoses	202843	10113657	52295188	69	27215
Hospital Encounters	145551	1163284	1163284	3	520
Lab Results	172795	2012185	58386934	84	27384
Ordered Medications	190256	3964120	15155203	23	7041
Microbiology Results	54798	145528	1988429	5	5174
Notes	204161	10014987	28938900	56	14933
Problem List	181221	N/A	1737749	6	204
Procedures Ordered	198833	5129756	19501225	31	35364
Result Comments	131104	896896	1542279	4	1765
Surgeries	44238	78403	83535	1	54
Vitals	199971	3500418	18268032	24	9442
Administered Medications	100565	349332	17160858	17	53178
Ambulatory Encounters	204235	12091755	12091755	27	1991

Table 4. Cross-validation performance of the final feature set on the entire data set for ranking the 30 confirmed cases of porphyria higher than the general population. SVM with radial basis function (RBF) kernel and gamma = 0.04.

Metric	Score
**AUC**	0.775
**Average Precision**	0.060
**Precision @ 100**	0.031
**Log Loss**	0.404

Table 5. Assessment of the likelihood of undiagnosed acute hepatic porphyria based on clinical note symptom documentation. Both groups of 100 reviewed patients are listed.

	**Acute Hepatic Porphyria?**	**# Patients**
No mention of porphyria group (n=100)	Diagnostic test is *Likely Indicated*	4
	Diagnostic test is *Possibly Indicated*	18
	Diagnostic test is *Unlikely Indicated*	68
	Deceased	10
'Porph' in clinical notes group (n=100)	Suspected in chart	16
	Suspected, ruled out in chart	15
	Diagnostic test is *Possibly Indicated*, not suspected in chart	4
	Unlikely based on chart review	54
	Diagnosed, documented in chart	4
	Unknown, unable to determine	1
	Deceased	6

Table 6. Top alternative explanations for AHP symptom profiles seen in both groups of patients. Conditions seen in no more than one patient are not listed.

	Alternate AHP Symptom Explanation	# Patients
No mention of porphyria group	Surgery	8
	Inflammatory Bowel Disease	6
	Cancer	6
	Cancer Chemotherapy	5
	Gallbladder Pathology	4
	Diabetes	3
	Carnitine Palmitoyl Transferase Deficiency	2
	Renal	4
	Poly Cystic Ovarian Syndrome	2
	Appendicitis	2
	Mastocytosis	2
'Porph' in clinical notes group	Liver Pathology	30
	Chemotherapy/Drug Side Effects	3
	Mastocytosis	2

Table 7. Age statistics in years for the two patient groups.

	NO MENTION OF PORPHYRIA	'PORPH' IN CLINICAL NOTES
**MEDIAN**	51	54
**MEAN**	53	50
**MIN**	8	6
**MAX**	91	91

Table 8. Sex distribution for the two patient groups.

	NO MENTION OF PORPHYRIA	'POPRH' IN CLINICAL NOTES
**MALE**	25	44
**FEMALE**	75	56

Table 9. Top reasons for the presence of the word ‘porph’ found in the clinical note.

More Common Reasons for 'Porph' in Clinical Notes	**# Patients**
Suspicion of Porphyria	31
Liver Transplant Documentation	30
Porphyria Mentioned in Treatment Precautions	18
Porphyria Diagnosis Mentioned in Notes	4
Porphyria Lab Tests Listed for Screening Physical	3
Family History of Porphyria	5
Misspelling	2

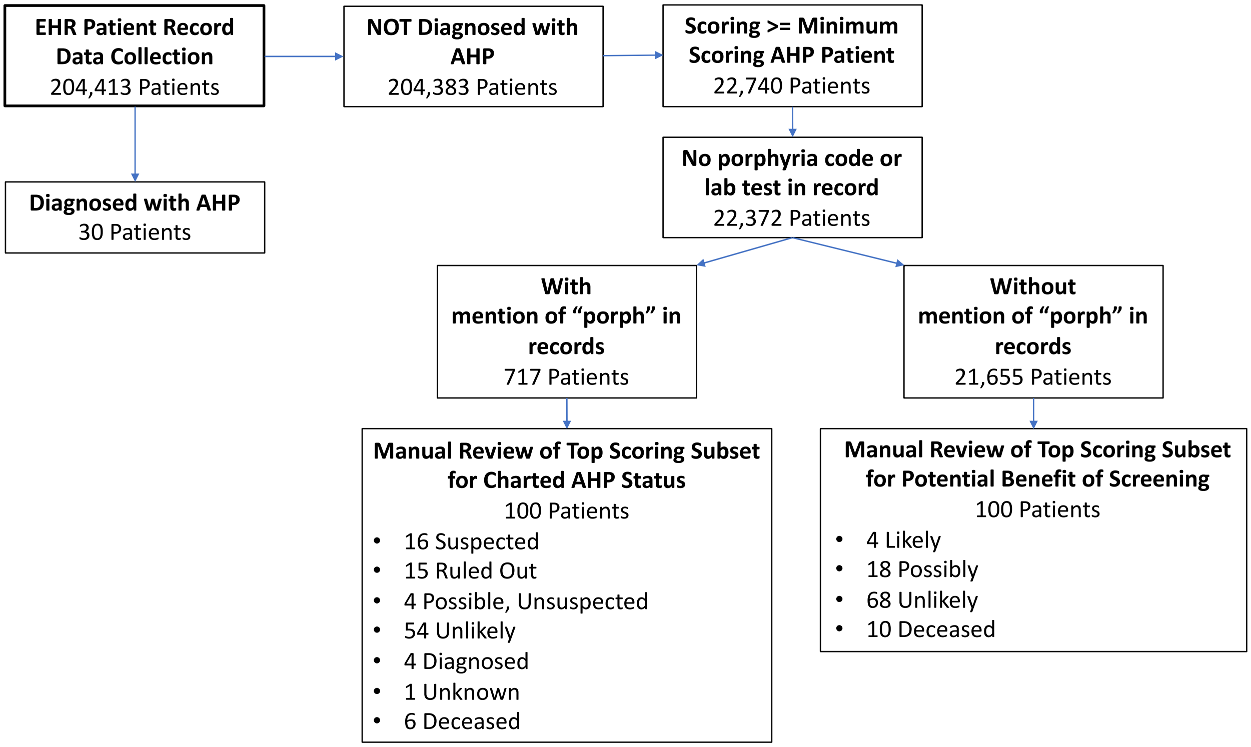

Figure 1. Flowchart of patient data record selection. Collection starts from full set of from full collection 204, 413 patient records and is filtered down to two sets of 100 records that were manually reviewed and characterized for 1) present indications for screening for AHP, and 2) status of AHP evaluation in the clinical notes of the record.

References

1. Meystre S, Lovis C, Bürkle T, Tognola G, Budrionis A, Lehmann C. Clinical data reuse or secondary use: current status and potential future progress. In: Holmes J, Soualmia L, Séroussi B, editors. Yearbook of Medical Informatics. 262017. p. 38-52.
2. Anonymous. Rare Diseases Act of 2002. Public Law 107 - 280; 2002 November 6, 2002.
3. Haendel M, Vasilevsky N, Unni D, Bologa C, Harris N, Rehm H, et al. How many rare diseases are there? Nature Reviews Drug Discovery. 2019.
4. Ramalle-Gómara E, Ruiz E, Quiñones C, Andrés S, Iruzubieta J, Gil-de-Gómez J. General knowledge and opinion of future health care and non-health care professionals on rare diseases. Journal of Evaluation in Clinical Practice. 2015;21:198-201.
5. Garg R, Dong S, Shah S, Jonnalagadda S. A bootstrap machine learning approach to identify rare disease patients from electronic health records. arXivorg. 2016:arXiv:1609.01586.
6. Colbaugh R, Glass K, Rudolf C, Tremblay M, editors. Learning to identify rare disease patients from electronic health records. AMIA Annual Symposium Proceedings; 2018; San Francisco, CA.
7. Shen F, Wang L, Liu H. Phenotypic analysis of clinical narratives using human phenotype ontology. Studies in Health Technology and Informatics. 2017;245:581-5.
8. Shen F, Liu S, Wang Y, Wen A, Wang L, Liu H. Utilization of electronic medical records and biomedical literature to support the diagnosis of rare diseases using data fusion and collaborative filtering approaches. JMIR Medical Informatics. 2018;6(4):e11301.
9. Besur S, Hou W, Schmeltzer P, Bonkovsky H. Clinically important features of porphyrin and heme metabolism and the porphyrias. Metabolites. 2014;4:977-1006.
10. Bissell D, Anderson K, Bonkovsky H. Porphyria. New England Journal of Medicine. 2017;377:862-72.
11. Gouya L, Ventura P, Balwani M, Bissell D, Rees D, Penz C, et al. EXPLORE: a prospective, multinational, natural history study of patients with acute hepatic porphyria with recurrent attacks. Hepatology. 2019:Epub ahead of print.
12. Ramanujam V, Anderson K. Porphyria diagnostics – Part 1: a brief overview of the porphyrias. Current Protocols in Human Genetics. 2015;86:17.20.1-17.20.6.
13. Szlendak U, Bykowska K, Lipniacka A. Clinical, biochemical and molecular characteristics of the main types of porphyria. Advances in Clinical and Experimental Medicine. 2016;25:361-8.
14. Elder G, Harper P, Badminton M, Sandberg S, Deybach J. The incidence of inherited porphyrias in Europe. Journal of Inherited Metabolic Disease. 2013;36:849-57.
15. Bonkovsky H, Maddukuri V, Yazici C, Anderson K, Bissell D, Bloomer J, et al. Acute porphyrias in the USA: features of 108 subjects from Porphyrias Consortium. American Journal of Medicine. 2014;127:1233-41.
16. Bonkovsky H, Dixon N, Rudnick S. Pathogenesis and clinical features of the acute hepatic porphyrias (AHPs). Molecular Genetics and Metabolism. 2019;128:213-8.
17. Chen B, Solis-Villa C, Hakenberg J, Qiao W, Srinivasan R, Yasuda M, et al. Acute intermittent porphyria: predicted pathogenicity of HMBS variants indicates extremely low penetrance of the autosomal dominant disease. Human Mutation. 2016;37:1215-22.
18. Anderson K, Bloomer J, Bonkovsky H, JP Kushner, Pierach C, Pimstone N, et al. Recommendations for the diagnosis and treatment of the acute porphyrias. Annals of Internal Medicine. 2005;142:439-50.

19. Pischik E, Kauppinen R. An update of clinical management of acute intermittent porphyria. The Application of Clinical Genetics. 2015;8:201-14.
20. Anonymous. PANHEMATIN® (hemin for injection) U.S. Prescribing Information. Recordati Rare Diseases. 2017:1-14.
21. Anonymous. Drug Trials Snapshots: GIVLAARI. Food & Drug Administration; 2019 November 20, 2019.
22. Sardh E, Harper P, Balwani M, Stein P, Rees D, Bissell D, et al. Phase 1 trial of an RNA interference therapy for acute intermittent porphyria. New England Journal of Medicine. 2019;380:549-58.
23. Anderson K. Porphyrias: An overview. Up To Date 2019.
24. Cherem J, Malagon J, Nellen H. Cimetidine and acute intermittent porphyria. Annals of Internal Medicine. 2005;143:694-5.
25. Cohen A, Smalheiser N, McDonagh M, Yu C, Adams C, Davis J, et al. Automated confidence ranked classification of randomized controlled trial articles: an aid to evidence-based medicine. Journal of the American Medical Informatics Association. 2015;22:707-17.
26. Sun Y, Liang D, Wang X, Tang X. Deepid3: Face recognition with very deep neural networks. arXivorg. 2015:arXiv:1502.00873.
27. Kaur H, Pannu H, Malhi A. A systematic review on imbalanced data challenges in machine learning: applications and solutions. ACM Computing Surveys (CSUR). 2019:79.
28. Dhar S, Cherkassky V. Development and evaluation of cost-sensitive universum-SVM. IEEE Transactions on Cybernetics. 2014;45:806-18.
29. Haixiang G, Yijing L, Shang J, Mingyun G, Yuanyue H, Bing G. Learning from class-imbalanced data: review of methods and applications. Expert Systems with Applications. 2017;73:220-39.

Supplemental Table 1. Final 141 features selected for inclusion in the machine learning model to predict acute hepatic porphyria. Features are scored by number of occurrances in an individual patient medical record, and then normalized.

INDEX	FEATURE	SOURCE DOCUMENTS	DESCRIPTION
1	ABDOMINAL_PAIN_DX_NAME	Encounter Diagnosis, Patient Problem List	Text description of diagnosis code (ICD9)
2	ABDOMINAL_PAIN_UNSPECIFIED_SITE_DX_NAME	Encounter Diagnosis, Patient Problem List	Text description of diagnosis code (ICD9)
3	ALTERNATIVE_THERAPY_-_PINEAL_HORMONE_AGENTS_PHARM_SUBCLASS_NAME	Concomittent Medications, Administered Medications, Medications Ordered	Text description of drug subclass
4	ANALGESIC_OPIOID_OXYCODONE_COMBINATIONS_PHARM_SUBCLASS_NAME	Concomittent Medications, Administered Medications, Medications Ordered	Text description of drug subclass
5	ANTI-ANXIETY_-_BENZODIAZEPINES_PHARM_CLASS_NAME	Concomittent Medications, Administered Medications, Medications Ordered	Text description of drug class
6	ANTICONVULSANT_-_GABA_ANALOGS_PHARM_SUBCLASS_NAME	Concomittent Medications, Administered Medications, Medications Ordered	Text description of drug subclass
7	ANTIEMETIC_-_PHENOTHIAZINES_PHARM_SUBCLASS_NAME	Concomittent Medications, Administered Medications, Medications Ordered	Text description of drug subclass
8	ANTIHISTAMINE_-_1ST_GENERATION_-_ETHANOLAMINES_PHARM_SUBCLASS_NAME	Concomittent Medications, Administered Medications, Medications Ordered	Text description of drug subclass
9	ANTIHISTAMINE_-_1ST_GENERATION_-_PHENOTHIAZINES_PHARM_SUBCLASS_NAME	Concomittent Medications, Administered Medications, Medications Ordered	Text description of drug subclass
10	BASO_#_COMPONENT_NAME	Lab Results	Percent Basophils performed

11	CALCIUM_REPLACEMENT_PHARM_CLASS_NAME	Concomittent Medications, Administered Medications, Medications Ordered	Text description of drug class
12	CBC_WITH_DIFFERENTIAL_PROC_NAME	Procedures Ordered	CBC with diff order present
13	CNSLT0031_PROC_CODE	Procedures Ordered	Code for consult to Gastroenterolo gy
14	CONSULT_TO_GASTROENTEROLOGY_PROC_NAME	Procedures Ordered	Consult to Gastoenterolog y ordered
15	COPD_(CHRONIC_OBSTRUCTIVE_PULMONARY_DISEASE)_(HCC)_ DX_NAME	Encounter Diagnosis, Patient Problem List	Text description of diagnosis code (ICD9)
16	CREATININE_URINE_CONCENTRATION_COMPONENT_NAME	Lab Results	lab result component present
17	CREATININEUR(REFERRAL)_COMPONENT_NAME	Lab Results	lab result component present
18	DIFFERENTIAL_PROC_NAME	Procedures Ordered	blood differential order present
19	DIPHENHYDRAMINE_HCL_GENERIC_NAME_1	Concomittent Medication, Medications Ordered	Generic name of medication
20	ELEVATED_WHITE_BLOOD_CELL_COUNT_UNSPECIFIED_DX_ICD 10_NAME	Encounter Diagnosis, Patient Problem List	Text description of diagnosis code (ICD10)
21	EOS_#_COMPONENT_NAME	Lab Results	eosinaphil count lab result present
22	ESSENTIAL_(PRIMARY)_HYPERTENSION_DX_ICD10_NAME	Encounter Diagnosis, Patient Problem List	Text description of diagnosis code (ICD10)
23	FERRITIN_SERUM_PROC_NAME	Procedures Ordered	serum ferritin order present
24	HYDROMORPHONE_HCL_GENERIC_NAME_1	Concomittent Medication, Medications Ordered	Generic name of medication
25	LAB00047_PROC_CODE	Procedures Ordered	Plasma lipase procedure ordered
26	LAB00364_PROC_CODE	Procedures Ordered	Microscopic urine exam ordered

27	LAB00681_PROC_CODE	Procedures Ordered	CBC with differential ordered
28	LAB100107_PROC_CODE	Procedures Ordered	Blood differential ordered
29	LAB100227_PROC_CODE	Procedures Ordered	Urine volume measurement ordered
30	LAB100882_PROC_CODE	Procedures Ordered	Multi-tube blood draw ordered
31	LIPASE__(LAB)_COMPONENT_NAME	Lab Results	plasma lipase result component present
32	LIPASE_PLASMA_PROC_NAME	Procedures Ordered	plasma lipase order present
33	LYMPHOCYTE_#_COMPONENT_NAME	Lab Results	blood lymphocyte count results present
34	MAGNESIUM_SALTS_REPLACEMENT_PHARM_CLASS_NAME	Concomittent Medications, Administered Medications, Medications Ordered	Text description of drug class
35	MELATONIN_GENERIC_NAME_1	Concomittent Medication, Medications Ordered	Generic name of medication
36	MINERALS_AND_ELECTROLYTES_-_CALCIUM_REPLACEMENT/VITAMIN_D_COMBINATIONS_PHARM_SUBCLASS_NAME	Concomittent Medications, Administered Medications, Medications Ordered	Text description of drug subclass
37	MISC_REF_TEST_NAME_COMPONENT_NAME	Lab Results	Special test given with name of test in RESULT_TEXT
38	MISC_REF_TEST_RESULT_COMPONENT_NAME	Lab Results	Result of special test present
39	MONOCYTE_#_COMPONENT_NAME	Lab Results	blood monocyte count results present
40	NAUSEA_WITH_VOMITING_UNSPECIFIED_DX_ICD10_NAME	Encounter Diagnosis, Patient Problem List	Text description of diagnosis code (ICD10)
41	NEUTROPHIL_#_COMPONENT_NAME	Lab Results	blood neutrophil count results present
42	NGRAM_0^pramipexole	Notes	Bigram of [token]^[token]

			found in free text.
43	NGRAM_0^tablet	Notes	Bigram of [token]^[token] found in free text.
44	NGRAM_10^olanzapine	Notes	Bigram of [token]^[token] found in free text.
45	NGRAM_10^tablet	Notes	Bigram of [token]^[token] found in free text.
46	NGRAM_100^sodium	Notes	Bigram of [token]^[token] found in free text.
47	NGRAM_4^mg	Notes	Bigram of [token]^[token] found in free text.
48	NGRAM_4^odt	Notes	Bigram of [token]^[token] found in free text.
49	NGRAM_90^albuterol	Notes	Bigram of [token]^[token] found in free text.
50	NGRAM_abdominal	Notes	Unigram of [token] found in free text.
51	NGRAM_abdominal^pain	Notes	Bigram of [token]^[token] found in free text.
52	NGRAM_acute	Notes	Unigram of [token] found in free text.
53	NGRAM_acute^distress	Notes	Bigram of [token]^[token] found in free text.
54	NGRAM_ambulatory	Notes	Unigram of [token] found in free text.
55	NGRAM_antibiotics	Notes	Unigram of [token] found in free text.
56	NGRAM_antibiotics^sulfonamide	Notes	Bigram of [token]^[token] found in free text.
57	NGRAM_atraumatic	Notes	Unigram of [token] found in free text.
58	NGRAM_bipolar	Notes	Unigram of [token] found in free text.

59	NGRAM_cigarettes	Notes	Unigram of [token] found in free text.
60	NGRAM_compazine	Notes	Unigram of [token] found in free text.
61	NGRAM_control^pain	Notes	Bigram of [token]^[token] found in free text.
62	NGRAM_depakote	Notes	Unigram of [token] found in free text.
63	NGRAM_dilaudid	Notes	Unigram of [token] found in free text.
64	NGRAM_discharged	Notes	Unigram of [token] found in free text.
65	NGRAM_disintegrating	Notes	Unigram of [token] found in free text.
66	NGRAM_docusate	Notes	Unigram of [token] found in free text.
67	NGRAM_docusate^sodium	Notes	Bigram of [token]^[token] found in free text.
68	NGRAM_dose^oral	Notes	Bigram of [token]^[token] found in free text.
69	NGRAM_duloxetine	Notes	Unigram of [token] found in free text.
70	NGRAM_ed	Notes	Unigram of [token] found in free text.
71	NGRAM_edisylate]	Notes	Unigram of [token] found in free text.
72	NGRAM_extended^tablet	Notes	Bigram of [token]^[token] found in free text.
73	NGRAM_fibromyalgia	Notes	Unigram of [token] found in free text.
74	NGRAM_flare	Notes	Unigram of [token] found in free text.
75	NGRAM_flares	Notes	Unigram of [token] found in free text.
76	NGRAM_focal	Notes	Unigram of [token] found in free text.
77	NGRAM_gallops	Notes	Unigram of [token] found in free text.

78	NGRAM_genitourinary	Notes	Unigram of [token] found in free text.
79	NGRAM_glycol	Notes	Unigram of [token] found in free text.
80	NGRAM_glycol^polyethylene	Notes	Bigram of [token]^[token] found in free text.
81	NGRAM_gram	Notes	Unigram of [token] found in free text.
82	NGRAM_hydromorphone	Notes	Unigram of [token] found in free text.
83	NGRAM_instructed	Notes	Unigram of [token] found in free text.
84	NGRAM_iv	Notes	Unigram of [token] found in free text.
85	NGRAM_latex	Notes	Unigram of [token] found in free text.
86	NGRAM_magnesium	Notes	Unigram of [token] found in free text.
87	NGRAM_melatonin	Notes	Unigram of [token] found in free text.
88	NGRAM_miralax	Notes	Unigram of [token] found in free text.
89	NGRAM_mouth^needed	Notes	Bigram of [token]^[token] found in free text.
90	NGRAM_mouth^twelve	Notes	Bigram of [token]^[token] found in free text.
91	NGRAM_nausea	Notes	Unigram of [token] found in free text.
92	NGRAM_nausea^vomiting	Notes	Bigram of [token]^[token] found in free text.
93	NGRAM_odt	Notes	Unigram of [token] found in free text.
94	NGRAM_odt^ondansetron	Notes	Bigram of [token]^[token] found in free text.
95	NGRAM_olanzapine	Notes	Unigram of [token] found in free text.

96	NGRAM_oncology	Notes	Unigram of [token] found in free text.
97	NGRAM_ondansetron	Notes	Unigram of [token] found in free text.
98	NGRAM_oral^powder	Notes	Bigram of [token]^[token] found in free text.
99	NGRAM_oxycodone	Notes	Unigram of [token] found in free text.
100	NGRAM_pain^severe	Notes	Bigram of [token]^[token] found in free text.
101	NGRAM_pathology	Notes	Unigram of [token] found in free text.
102	NGRAM_penicillins	Notes	Unigram of [token] found in free text.
103	NGRAM_phenergan	Notes	Unigram of [token] found in free text.
104	NGRAM_polyethylene	Notes	Unigram of [token] found in free text.
105	NGRAM_powder	Notes	Unigram of [token] found in free text.
106	NGRAM_pramipexole	Notes	Unigram of [token] found in free text.
107	NGRAM_propranolol	Notes	Unigram of [token] found in free text.
108	NGRAM_protocol	Notes	Unigram of [token] found in free text.
109	NGRAM_psychosis	Notes	Unigram of [token] found in free text.
110	NGRAM_risperidone	Notes	Unigram of [token] found in free text.
111	NGRAM_rubs	Notes	Unigram of [token] found in free text.
112	NGRAM_scoliosis	Notes	Unigram of [token] found in free text.
113	NGRAM_seroquel	Notes	Unigram of [token] found in free text.
114	NGRAM_severe	Notes	Unigram of [token] found in free text.

115	NGRAM_stomach	Notes	Unigram of [token] found in free text.
116	NGRAM_sulfa	Notes	Unigram of [token] found in free text.
117	NGRAM_sulfonamide	Notes	Unigram of [token] found in free text.
118	NGRAM_urine	Notes	Unigram of [token] found in free text.
119	NGRAM_vicodin	Notes	Unigram of [token] found in free text.
120	NGRAM_zofran	Notes	Unigram of [token] found in free text.
121	NORMAL_RANGE_COMPONENT_NAME	Lab Results	Lab test result within normal ranges
122	OBSTRUCTIVE_SLEEP_APNEA_(ADULT)_(PEDIATRIC)_DX_ICD10_NAME	Encounter Diagnosis, Patient Problem List	Text description of diagnosis code (ICD10)
123	OBSTRUCTIVE_SLEEP_APNEA_DX_NAME	Encounter Diagnosis, Patient Problem List	Text description of diagnosis code (ICD9)
124	ONDANSETRON_HCL_GENERIC_NAME_1	Concomittent Medication, Medications Ordered	Generic name of medication
125	OXYCODONE_HCL/ACETAMINOPHEN_GENERIC_NAME_1	Concomittent Medication, Medications Ordered	Generic name of medication
126	PATHOLOGY_PROC_NAME	Procedures Ordered	Transcribed pathology report present
127	PELVIC_AND_PERINEAL_PAIN_DX_ICD10_NAME	Encounter Diagnosis, Patient Problem List	Text description of diagnosis code (ICD10)
128	PINEAL_HORMONE_AGENTS_PHARM_CLASS_NAME	Concomittent Medications, Administered Medications, Medications Ordered	Text description of drug subclass
129	PROCHLORPERAZINE_EDISYLATE_GENERIC_NAME_1	Concomittent Medication, Medications Ordered	Generic name of medication
130	PROMETHAZINE_HCL_GENERIC_NAME_1	Concomittent Medication, Medications Ordered	Generic name of medication

131	RADIOLOGY_PROC_NAME	Procedures Ordered	Transcribed radiology report present
132	RAINBOW_HOLD_TUBE_-_BLUE_TOP_PROC_NAME	Procedures Ordered	Multi-tube blood draw ordered
133	RESTLESS_LEGS_SYNDROME_DX_ICD10_NAME	Encounter Diagnosis, Patient Problem List	Text description of diagnosis code (ICD10)
134	TOBACCO_ABUSE_DX_NAME	Encounter Diagnosis, Patient Problem List	Text description of diagnosis code (ICD9)
135	TRIPLE_P04_CRYSTALS_COMPONENT_NAME	Lab Results	Component of result of lab test
136	TRNS00039_PROC_CODE	Procedures Ordered	Transcribed pathology report present
137	TRNS00040_PROC_CODE	Procedures Ordered	Transcribed imaging report present
138	UNSPECIFIED_ABDOMINAL_PAIN_DX_ICD10_NAME	Encounter Diagnosis, Patient Problem List	Text description of diagnosis code (ICD10)
139	UNSPECIFIED_ABDOMINAL_PAIN_DX_ICD10_NAME	Encounter Diagnosis, Patient Problem List	Text description of diagnosis code (ICD10)
140	URINE_MICROSCOPIC_EXAM_PROC_NAME	Lab Results	Name of lab test procedure
141	VOL(URINE)_PROC_NAME	Lab Results	Name of lab test procedure

Tracked Updated Manuscript

Detecting Rare Diseases in Electronic Health Records Using Machine Learning and Knowledge Engineering: Case Study of Acute Hepatic Porphyria

Aaron Cohen, MD, MS [1*]
Steven Chamberlin, ND [1]
Thomas Deloughery, MD [1]
Michelle Nguyen, BS [1]
Steven Bedrick, PhD [1]
Stephen Meninger, PharmD [2]
John J. Ko, PharmD, MS [2]
Jigar Amin, PharmD [2]
Alex Wei, PharmD [2]
William Hersh, MD [1]

[1]Department of Medical Informatics & Clinical Epidemiology, School of Medicine, Oregon Health & Science University, Portland, OR USA.

[2]Alnylam Pharmaceuticals, Cambridge, MA, USA.

* Corresponding Author:
Aaron M. Cohen, MD MS
Professor
Department of Medical Informatics & Clinical Epidemiology
School of Medicine
Oregon Health & Science University
Portland, Oregon USA 97239
Email: cohenaa@ohsu.edu

Abstract

Background

With the growing adoption of the electronic health record (EHR) worldwide over the last decade, new opportunities exist for leveraging EHR data for detection of rare diseases. Rare diseases are often not diagnosed or delayed in diagnosis by clinicians who encounter them infrequently. One such rare disease that may be amenable to EHR-based detection is acute hepatic porphyria (AHP). AHP consists of a family of rare, metabolic diseases characterized by potentially life-threatening acute attacks and, for some patients, chronic debilitating symptoms that negatively impact daily functioning and quality of life. The goal of this study was to apply machine learning and knowledge engineering to a large extract of EHR data to determine whether they could be effective in identifying patients not previously tested for AHP who should receive a proper diagnostic workup for AHP.

Methods and Findings

We used an extract of the complete EHR data of 200,000 patients from an academic medical center for up to 10 years longitudinally and enriched it with records from an additional 5,571 patients from the center containing any mention of porphyria in notes, laboratory tests, diagnosis codes, and other parts of the record. After manually reviewing the records of all 47 unique patients with the ICD-10-CM code E80.21 (Acute intermittent [hepatic] porphyria), we identified 30 patients who were positive cases for our machine learning models, with the rest of the patients used as negative cases. We parsed the record into features, which were scored by frequency of appearance and labeled by the EHR source document. We then carried out a univariate feature analysis, manually choosing features not directly tied to provider attributes or suspicion of the patient having AHP. We next trained on the full dataset, with the best cross-validation performance coming from support vector machine (SVM) algorithm using a radial basis function (RBF) kernel. The trained model was applied back to the full data set and patients were ranked by margin distance. The top 100 ranked negative cases were manually reviewed for symptom complexes similar to AHP, finding four patients where AHP diagnostic testing was likely indicated and 18 patients where AHP diagnostic testing was possibly indicated. From the top 100 ranked cases of patients with mention of porphyria in their record, we identified four patients for whom AHP diagnostic testing was possibly indicated and had not been previously performed. Based solely on the reported prevalence of AHP, we would have expected only 0.002 cases out of the 200 patients manually reviewed.

Conclusions

The application of machine learning and knowledge engineering to EHR data may facilitate the diagnosis of rare diseases such as AHP. The only manual modifications to this work were the removal of disease-specific or medical center specific features that might undermine our ability to find new cases. Further work will recommend clinical investigation to identified patients' clinicians, evaluate more patients, assess additional feature selection and machine learning algorithms, and apply this methodology to other rare diseases.

Introduction

The growing adoption of the electronic health record (EHR) worldwide has created new opportunities for leveraging EHR data for other, so called *secondary* purposes, such as clinical and translational research, quality measurement and improvement, patient cohort identification and more {Meystre, 2017 #10530}. One emerging use case for leveraging of EHR data is to detect undiagnosed rare diseases. Although there is no absolute definition of a rare disease, the US Rare Diseases Act of 2002 defines rare diseases as those that occur in fewer than 200,000 patients worldwide {Anonymous, 2002 #11601}, and the National Organization for Rare Disorders (NORD, https://rarediseases.org/) registry lists more than 1,200 diseases. Others have noted that the true number of rare diseases is unknown, and have called for more research to define them {Haendel, 2019 #11646}.

Rare diseases can be difficult to diagnose because their infrequent occurrence may result in primary care physicians not considering them in diagnostic workups {Ramalle-Gómara, 2015 #12199}. They also often have general presentations with diffuse symptoms, as well as genetic components which may require specialized testing. This lack of timely diagnosis may lead to both physical and emotional suffering as patients remain undiagnosed for prolonged periods. Additionally, a lack of accurate diagnoses increases economic burden to healthcare systems as patients continue to receive inadequate and/or inappropriate treatment. Some informatics researchers have used EHR data to detect rare diseases, such as cardiac amyloidosis {Garg, 2016 #11604}, lipodystrophy {Colbaugh, 2018 #11605}, and a large collection of different diseases {Shen, 2017 #11607;Shen, 2018 #11606}.

One rare disease that may be amenable to EHR-based detection is acute hepatic porphyria (AHP). AHP is a subset of porphyria that refers to a family of rare, metabolic diseases characterized by potentially life-threatening acute attacks and, for some patients, chronic debilitating symptoms that negatively impact daily functioning and quality of life {Besur, 2014 #11907;Bissell, 2017 #11905;Gouya, 2019 #11908;Ramanujam, 2015 #11904;Szlendak, 2016 #11906}. During attacks, patients typically present with multiple signs and symptoms due to dysfunction across the autonomic, central, and peripheral nervous systems. The prevalence of diagnosed symptomatic AHP patients is ~1 per 100,000 {Elder, 2013 #11603}. Due to the nonspecific symptoms and the rare nature of the disease, AHP is often initially overlooked or misdiagnosed. A U.S. study demonstrated that diagnosis of AHP is delayed on average by up to 15 years {Bonkovsky, 2014 #11659}.

AHP is predominantly caused by a genetic mutation leading to a partial deficiency in the activity of one of the eight enzymes responsible for heme synthesis {Ramanujam, 2015 #11904}. These defects predispose patients to the accumulation of neurotoxic heme intermediates aminolevulinic acid (ALA) and porphobilinogen (PBG) when the rate limiting enzyme of the heme synthesis pathway, aminolevulinic acid synthase 1 (ALAS1), is induced {Bissell, 2017 #11905;Bonkovsky, 2019 #11909}. Gene mutations causing the disease are mostly autosomal dominant, however the disease has low penetrance (~1%) and many specific mutations have not been identified {Chen, 2016 #11910}. Furthermore, families carrying the gene may have few or only one affected member. Therefore, family history can be a poor diagnostic tool for this

disease. The preferred diagnostic procedure for AHP is biochemical testing of random/spot urine for ALA, PBG, and porphyrins {Anderson, 2005 #11911;Pischik, 2015 #11912}.

Historically, treatment of AHP has predominantly focused on avoidance of attack triggers, management of pain and other chronic symptoms, and treatment of acute attacks through the use of Panhematin® (hemin for injection) {Anonymous, 2017 #11913}. Panhematin was FDA approved in 1983 for the amelioration of recurrent attacks of acute intermittent porphyria (AIP) temporally related to the menstrual cycle in susceptible women after initial carbohydrate therapy is known or suspected to be inadequate ~~{Anonymous, 2017 #11913}~~.

Recently, a new drug Givlaari® (givosiran), for subcutaneous injection has been approved by the FDA for the treatment of adults with AHP {Anonymous, 2019 #11914}. Givosiran is a double-stranded small interfering RNA (siRNA) molecule that reduces induced levels of the protein ALAS1. A Phase 1 trial has been published {Sardh, 2019 #11562} and a Phase 3 randomized control trial has shown this therapy to be effective in reducing the occurrence of acute attacks and impacting other manifestations of the disease {Anonymous, 2019 #11914}.

~~Oregon Health & Science University (OHSU) is the only academic medical center in Oregon and is thus a referral center for rare diseases like AHP. The OHSU Research Data Warehouse (RDW) is a research data "honest broker" service that provides EHR data to researchers, with appropriate IRB approval. The investigators have an ongoing institutional review board (IRB) approval to use an extract from the Oregon Health & Science University (OHSU) EHR research data warehouse (RDW) for a series of patient cohort identification projects. For this research, the patient cohort to identify was defined as those patients who have a documented clinical history of AHP, or a clinical history indicating that AHP diagnostic testing may be appropriate. The goal of this study was to apply machine learning and knowledge engineering to a large extract of EHR data to determine whether the combined approach could be effective in identifying patients not previously tested for AHP who should receive a proper diagnostic workup for AHP.~~

Materials and Methods

This study protocol was approved by the OHSU Institutional Review Board (IRB00011159).

Dataset

Oregon Health & Science University (OHSU) is the only academic medical center in Oregon and is thus a referral center for rare diseases like AHP. The OHSU Research Data Warehouse (RDW) is a research data "honest broker" service that provides EHR data to researchers, with appropriate IRB approval. The investigators have an ongoing institutional review board (IRB) approval to use an extract from the Oregon Health & Science University (OHSU) EHR research data warehouse (RDW) for a series of patient cohort identification projects. For this research, the patient cohort to identify was defined as those patients who have a documented clinical history of AHP, or a clinical history indicating that AHP diagnostic testing may be appropriate. The goal of this study was to apply machine learning and knowledge engineering to a large extract of EHR data to determine whether the combined approach could be effective in identifying patients not previously tested for AHP who should receive a proper diagnostic workup for AHP.

Commented [AMC1]: Revise or keep?

Reviewer comment:
While this is important background information, it's not clear if this paragraph is needed in the paper, other than noting the diagnostic/prognostics should rely on biomarker and other lab tests rather than family history. Consider removing, or condensing

Currently our response is to keep the text and add to the cover letter:
This text is important to provide the patient disease context for our work, and provides a bit of additional clinical and genetic background to orient readers who may not have the detailed expertise about this disease, such as informaticians and machine learning researchers. The difficult diagnosis of AHP is in part due to the disease low penetrance and inconsistent appearances in families even though AHP and related diseases are mostly autosomal dominant.

Formatted: Normal

A large dataset of approximately 200,000 patient records was requested from the RDW, complete as of the data pull date in March 2019, including over 30 million text notes plus other document types. The data set goes back to the start of OHSU using ~~our current~~the Epic EHR system~~, xx xxx xxx~~ in January, 2009. These records ~~corresponded to~~consist of all patients who had more than one primary care health care visit at our institution. Each patient record was represented as a collection of documents of types given in **Table 1**. Patient records could include zero or more documents of each type.

> **Commented [AMC2]:** Bill do you have a date we can add here?

> **Commented [AMC3]:** Bill do you have a date here or can you revise this sentence?

To insure an adequate ~~number of number of patients~~sample size to make predictive models robust, we enriched the data set for possible AHP by adding records from an additional 5,571 patients who met one or more of the following case-insensitive criteria (see **Table 2**):

- Diagnosis including the wildcard search term "porph*" in the diagnosis name
- Medication including the wildcard search term "hemin*" in the medication name
- Procedure including the wildcard search term "porph*" in the procedure name
- Clinical or result note including the wildcard search term "porph*" in the note text

To develop a gold standard for the data, a medical student (MN), overseen by clinical experts among the rest of the authors, conducted a chart review to ~~identified~~ identify patients with a ~~high likelihood~~ confirmed diagnosis of AHP. We manually reviewed all the patients with the ICD-10-CM code E80.21 (Acute intermittent [hepatic] porphyria) in their record, looking for positive confirmation of AHP either through a lab test or a specific comment in a progress note. This process yielded 30 positive cases from the 47 coded for E80.21. As OHSU is the only academic medical center in Oregon and is thus a referral center for rare diseases like AHP, this may explain why the number of identified AHP patients in our database was higher than that which would be expected based on the global prevalence of AHP. For the remaining 17 records, we could not confirm by chart review the diagnosis of AHP. This may be due to the code being attached to the patient based on an encounter to rule out AHP, inaccurate past medical history data, or a charting error. For these 17 patients no additional information supporting the AHP diagnosis was found in the notes, clinical tests or medication records and the only evidence of AHP was an ICD-10-CM code at one place in the medical record.

The rest of the records were then assumed to be negative for AHP for the purposes of statistical analysis and machine learning. The data set consisted of the positive records plus the presumed negative records. The entire data set was used for statistical analysis and training the machine learning models, the final goal of which was to identify the presumed negative records which are actually likely to be positive.

We then deconstructed each patient record into a number of features to be used for machine learning. Structured data fields were encoded directly with the entire field content used as the feature.~~.~~ Free-text fields were parsed into unigrams and bigrams.~~.~~

All features were labeled with their source document fields. This enabled, for example, diagnosis names in ICD-10-CM code fields~~s~~ in the problem list to be distinguished from the same ~~ICD-10-CM codes appearing in an encounter diagnosis~~text appearing in free text notes. Feature values were encoded as the number of occurrences in the entire record for the patient. A summary of the types and counts of documents in the data set is shown in **Table 3**.

> **Commented [AMC4]:** Needed to fix this because what we said before was not quite correct.

Machine Learning Model Feature Selection and Training

Feature Selection and Machine Learning Methods

Features to be included in the machine learning model were selected by performing univariate logistic regression analysis of the entire feature set, using the confirmed AHP patients as positive samples and the rest of the data set as negative samples. For each document type, the 100 top features were chosen, ranked by odds ratio, having a p-value < 0.01 and occurring in at least 4 positive case patient records. This statistical criteria was used to establish which data elements had a significant relationship between the outcome variable, which was the presence, or not, of a confirmed diagnosis of AHP. Requiring that included features have at least four positive case patient records was chosen as a filter to strike a balance between only keeping the most common features, and keeping thousands of rare features requiring manual review that were unlikely be helpful in a generalized model.

From these several hundred features, a manual review process was performed to ensure that none of these features were directly connected to a diagnosis of AHP, mention of AHP in the record, or treatment of AHP. This was done by inspection. This process eliminated all text features mentioning any bigram of “acute hepatic porphyria,” medications such as hematin, and laboratory codes that in the OHSU system represented tests specifically for the diagnosis of porphyria.

The remaining features were then evaluated by using them in a machine learning model and scoring the model using 5 repetitions of 2-fold cross-validation. Several SVM kernel functions were tested including linear, polynomial degree 2, and the radial basis function (RBF), random forests, Adaboost, J48, and several topologies of Neural Network. Two normalization encoding methods were tried as well, binary, linear and log normalizing feature occurance counts between 0.0 and 1.0.

After algorithm selection, a second round of feature screening was performed. Any features with non-zero algorithm weights were removed if any direct connection to AHP could be established. This was performed by close scrutiny and discussion with our clinical expert for each feature. This second pass incorporated a higher level of clinical expertise than the first pass. It was performed after filtering by machine learning weights in order to reduce the screening load on our clinical expert.

Features to be included in the machine learning model were then selected by performing univariate analysis of the entire feature set, using the confirmed AHP patients as positive samples and the rest of the data set as negative samples. For each document type, the 100 top features were chosen, ranked by odds ratio, having a p-value < 0.01 and occurring in at least 4 positive case patient records.

From these several hundred features, a manual review process was performed to ensure that none of these features were directly connected to a diagnosis of AHP, mention of AHP in the record, or treatment of AHP. This process eliminated all text features mentioning any bigram of “acute hepatic porphyria,” medications such as hematin, and laboratory codes that in the OHSU system represented tests specifically for the diagnosis of porphyria.

Commented [AMC5]: The Materials and Methods section requires considerable revision. Please only report the methods employed to study the hypothesis of the study- results from any analyses, including model building and sensitivity analyses, should be reported in the Results section. The outcome variable is not clearly defined, although the authors do note they rank features but univariate odds ratios, which I assume are represent the likelihood of a patient being diagnosed for AHP.
The methods should include clear rationale for why a particular method was employed. If experiments are performed to further refine a model, the methods should be stated in this section, followed by the results of the methods in the results section. Machine Learning methods can be iterative, and may require manual review and revisions for model building, but this should be clearly outlined in the methods (e.g. how and why it is applied to the data

This process reduced the set to approximately 200 features. These features were then evaluated by using them in a machine learning model and scoring the model using 5 repetitions of 2-fold cross-validation. These experiments found that an SVM with the radial basis function (RBF) kernel scored best for the ranking metrics AUC and average precision. Linear SVM, random forests, Adaboost, J48, and several topologies of Neural Network were also tried but failed to perform as well as the RBF SVM. It was also determined that feature values were best encoded using log normalization, transforming feature occurrence counts into values between 0.0 and 1.0. Binary encoding, as well as linear normalization, failed to perform as well. We used the SVMLight implementation of the RBF kernel. Experimentation with cross-validation showed gamma = 0.04 to be optimal.

After algorithm selection, a second round of feature screening was performed. Any features with non-zero weights in the SVM model were removed if any direct connection to AHP could be established. This was performed by close scrutiny and discussion with clinical experts on each feature. For example, based on case series evidence, clinical hematology AHP specialists sometimes use cimetidine to treat AHP symptoms, as it is known to block a portion of the heme synthesis pathway as a side effect {Cherem, 2005 #11660}. We found that cimetidine was a highly weighted feature in our initial models (due to its use by a specialist [TD] at OHSU based on case report data {Cherem, 2005 #11660}) that had to be removed as it is given in response to AHP rather than being predictive. This process resulted in 146 total features being included in the final model.

The 146 features included in the final model are shown in **Table S-1**. Final feature set cross-validation performance on the entire training set is shown in **Table 4**.

Commented [AMC6]: Move to results, not methods

Machine Learning for AHP Prediction and Evaluation Methodology

A final trained model using the features selected was created by training the selected algorithm with chosen parameter settingsmode on the entire data set. This model was then applied back to the entire data set in order to create an AHP prediction score for each patient. The classifier margin distance was taken as the prediction score.

The patient prediction scores were then analyzed. To keep the manual chart review process manageable, we could not review every patient. In particular, the range of scores obtained for the 30 confirmed positive training cases were compared to the rest of the patients in the data set. About 22,000 patients in the general population had scores that overlapped with those of the 30 positive patients. While this was only 10% of the patient records, it was more than could be manually reviewed. We decided to review the top scoring 100 cases manually from each of two subsets of the general population.

The first reviewed subset of 100 patients were those with no mention of porphyria in their chart, no related ICD-9-CM or ICD-10-CM codes, and no porphyria specific lab test. We selected the top scoring 100 patients that met these criteria. This represents the most important target population for our project – patients with persistent symptoms that have not had AHP considered and tested to rule it in or out as a diagnosis. Manual review of these cases is intended to demonstrate the potential of our proposed approach to identify potential cases of AHP that would benefit from diagnostic testing and follow up.

The second reviewed subset of 100 patients were those with a mention of porphyria in the text notes in their chart, but no related ICD-9-CM or ICD-10-CM diagnosis codes, and no porphyria-

specific lab test. These are patients where porphyria may have been considered by the clinician, or may have been tested at another health care facility with unavailable records, or may have been a work up in progress. Manual review of these cases was intended to discern the clinical face validity of the algorithmic predictions, that is, the high scoring patients in this group score high because the algorithm is paying attention to some of the same non-AHP-specific clinical symptoms and other variables as the clinician. While the manual review of these patients was primarily intended for gaining insight into how the algorithm was scoring patients with porphyria mentioned in the charts, based on the manual review some patients who may benefit from diagnostic testing could be found.

A clinically trained reviewer assessed the patients' records in these two non-overlapping subsets for symptom patterns consistent with acute hepatic porphyria (AHP). The reviewer was blinded to the model features. Clinical notes were searched for the 'classic triad' of AHP symptoms: abdominal pain, central nervous system abnormalities, and peripheral neuropathy {Anderson, 2019 #11643}. In addition, any report of pain was assessed, and searches were also conducted for the highest incident AHP symptoms: abdominal pain, vomiting, constipation, muscle weakness, psychiatric symptoms, limb, head, neck, or chest pain, hypertension, tachycardia, convulsion, sensory loss, fever, respiratory paralysis, diarrhea {Anderson, 2019 #11643}. All major comorbidities were also reviewed and documented, as well as alternative diagnoses to explain AHP symptom profiles.

The 100 patients with no mention of porphyria in their EHR record were classified into one of three categories: *AHP diagnostic testing likely indicated, AHP diagnostic testing possibly indicated,* and *AHP diagnostic testing unlikely indicated.* To be classified as *likely*, symptoms had to be present in all three categories of the 'classic triad', without a cause identified in the EHR, and with a substantial history of symptoms. To be classified as *possibly*, symptoms had to be present in at least one of the three categories, without a cause documented and with a substantial history. Patients were classified as *unlikely* if their symptoms could be explained by another diagnosis, or if they did not have a strong AHP symptom profile.

The 100 patients who did have a mention of porphyria in their clinical notes were classified into one of five categories of AHP status based on chart review and details in the clinical notes: *AHP already suspected, AHP already suspected but ruled out*, *diagnostic testing likely indicated but AHP not suspected*, *unlikely AHP*, and *AHP diagnosis mentioned in notes*. A patient was classified as *AHP already suspected* if there was any level of AHP suspicion mentioned in their clinical notes, without a formal diagnosis or lab test. *AHP already suspected but ruled out* was assigned if there was a suspicion of AHP in the note, but had been ruled out, usually by negative lab tests. These lab tests were only documented in the note, since we excluded patients from this subset who had lab tests in the laboratory data itself. *Diagnostic testing likely indicated but AHP not suspected* was assigned if there were symptoms present in at least one of the three triad categories, without a cause, but no suspicion of AHP mentioned in the notes. For these patients the clinical notes contained the string 'porph' but presence of 'porph' in the clinical note was not related to suspicion of AHP. *Unlikely AHP* was assigned if AHP type symptoms could be explained by another diagnosis, or there was not a strong AHP symptom profile. Finally, patients were assigned to *AHP diagnosis* if there was any mention of an existing AHP diagnosis in the notes, even patient reported. The reasons for the presence of the string 'porph' in the clinical note for the second set of 100 patients was also reviewed and documented. Patient's categorized as *AHP already suspected* and *Diagnostic testing likely indicated but AHP not suspected* would

benefit from AHP testing as they displayed suspicion of AHP or symptom complexes associated with AHP but have yet received a full diagnostic work-up.

~~Figure 1 shows a flowchart of the overall patient record filtering and manual review process. The process starts with 204,413 patient records, and using a combination of machine learning and structured data filtering described above, identifies 200 patients that were manually reviewed. 100 of those patients were identified as not having any mention of porphyria in the medical record and potentially could benefit from AHP diagnostic testing. The other 100 of those patients did have mention of porphyria in their medical record, but no diagnostic code for porphyria. These records were reviewed to determine the reason for the mention of porphyria and evaluate whether these reasons were consistent with the goal of the machine learning to identify patients with symptoms and other clinical features consistent with a possible porphyria diagnosis.~~

Commented [AMC7]: Move to beginning of the results section

Results

Final selected features and machine learning cross-validation

Formatted: Font: Italic

Figure 1 shows a flowchart of the overall patient record filtering and manual review process. The process starts with 204,413 patient records, and using a combination of machine learning and structured data filtering described above, identifies 200 patients that were manually reviewed. 100 of those patients were identified as not having any mention of porphyria in the medical record and potentially could benefit from AHP diagnostic testing. The other 100 of those patients did have mention of porphyria in their medical record, but no diagnostic code for porphyria. These records were reviewed to determine the reason for the mention of porphyria and evaluate whether these reasons were consistent with the goal of the machine learning to identify patients with symptoms and other clinical features consistent with a possible porphyria diagnosis.

Commented [AMC8]: Move to beginning of the results section

Several hundred features made it through the statistical testing and occurrence frequency filter. From these several hundred features, the manual review process reduced the set to approximately 200 features. These features were then evaluated by using them in a machine learning model and scoring the model using 5 repetitions of 2-fold cross-validation. These experiments found that an SVM with the radial basis function (RBF) kernel scored best for the ranking metrics AUC and average precision. The other machine learning methods explored failed to perform as well as the RBF SVM. It was also determined that feature values were best encoded using log normalization, transforming feature occurrence counts into values between 0.0 and 1.0. Binary encoding, as well as linear normalization, failed to perform as well. We used the SVMLight implementation of the RBF kernel. Experimentation with cross-validation showed gamma = 0.04 to be optimal.

Formatted: Normal

After algorithm selection and tuning, the second round of feature screening removed a few features that the SVM model assigned non-zero weights which were thought to be directly connected to the pre-established diagnosis of AHP by the clinical expert. For example, based on case series evidence, clinical hematology AHP specialists sometimes use cimetidine to treat AHP symptoms, as it is known to block a portion of the heme synthesis pathway as a side effect {Cherem, 2005 #11660}. We found that cimetidine was a highly weighted feature in our initial models (due to its use by a specialist [TD] at OHSU based on case report data {Cherem, 2005 #11660}) that had to be removed as it is given in response to AHP rather than being predictive. This process resulted in 141 total features being included in the final model.

The 141 features included in the final model are shown in **Table S-1**. Final feature set cross-validation performance on the entire training set is shown in **Table 4.**

Commented [AMC9]: Move to results, not methods

Application of machine learning to the full data set

Formatted: Font: Italic

The final machine learning model with the 141 features was trained on the entire data set, and this model was then applied back to the entire data set in order to provide a margin distance score for every patient.

The patient prediction scores were then analyzed. In particular, the range of scores obtained for the 30 confirmed positive training cases were compared to the rest of the patients in the data set. About 22,000 patients in the general population had scores that overlapped with those of the 30 positive patients. While this was only 10% of the patient records, it was more than could be manually reviewed.

We reviewed the top scoring 100 cases manually from each of two subsets of the general population. Out of the 100 patient charts we reviewed with no mention of porphyria, four were identified as likely to *AHP diagnostic testing likely indicated*, all without mention of porphyria in their medical record or documentation of a urine PBG test. The first patient was a male with six years of unexplained intermittent abdominal pain with nausea, vomiting, and diarrhea. His other conditions included complex regional pain syndrome, peripheral neuropathy, cardiac arrhythmias, panic attacks, and depression. The next patient was a female whose abdominal pain was described as 'a long standing symptom with extensive negative evaluation'. Also listed in her profile were neuralgias, hereditary small fiber neuropathy, movement disorder, fibromyalgia, migraines, palpitations, and somatization disorder. The third patient was a woman with multiple emergency department admissions for severe abdominal pain. She also had severe suicidality with a permanent tracheostomy due to a hanging attempt, borderline personality disorder, tachycardia, anxiety, saddle anesthesia, insomnia, and severe somatization disorder including a comment in her note advising not to admit the patient for only vague complaints. The fourth patient was a female with a history of abdominal pain comments in the notes describing that the etiology had not been identified for her complex symptomology which included headaches, abdominal pain, paresthesias and palpitations.

Formatted: Paragraph

Overall, about a quarter of the 100 patients in the group without mention of porphyria had symptom profiles that were consistent with undiagnosed AHP and AHP diagnostic testing would either be likely or possibly indicated (**Table 5**). In this group there was no sign or suspicion of AHP by the clinician in the record. This is a much higher concentration of possible AHP patients than would be expected by chance based on the known prevlance of AHP.

Alternate explanations for characteristic AHP symptom profiles were diverse in the patient group without any mention of porphyria (**Table 6).** Cancers seen in this group included breast, uterine, pancreatic, cervical, leukemia and adrenal carcinoma. Other common comorbidities and conditions seen in this group included: fibromyalgia, irritable bowel syndrome, chronic fatigue, obesity, hypertension, obstructive sleep apnea, and chronic obstructive pulmonary disease. In contrast, alternate symptom profiles in the group with mention of porphyria in the notes were dominated by liver pathologies, mostly hepatocellular carcinoma.

Patients in the group *without* mention of porphyria in the medical record generally had much longer and more complicated histories compared to the other group, with 86 out of 100 having encounters spread over four years or longer. The patients *with* porphyria mentioned in the clinical notes tended to have shorter, and less complex histories (only 39 out of 100 had over 4 years of encounters), more focused on a single medical issue or set of symptoms, which may have been due to their being referral to our academic medical center from other health care sites.

There were small differences in age summary statistics between the two groups (**Table 7**), but notably more pediatric patients in the reviewed group with mention of porphyria found in clinical notes than those without (10 patients vs 1 patient). There were significantly more male patients found in this group too, compared to the group with no mention of porphyria (**Table 8**). Associated conditions for these 44 male patients were dominated by only a few diagnoses/symptom patterns: liver disease (N=18), suspicion of porphyria (N=11), or actinic keratosis (N=3). In contrast, no single condition dominated the male disease distribution in the patient group without mention of porphyria in the notes.

About a third of patients in the group *with* mention of porphyria in the clinical notes had some level of suspicion and work-up for AHP documented. We also identified four patients in this group that we thought had possibly undiagnosed AHP, without suspicion documented in the notes. We labeled these patients as *Diagnostic testing likely indicated but AHP not suspected.* Three of these patients had 'porphyria' in their clinical note listed as a standard precaution for several different medications (hydrochloroquinone, ferrous sulfate), which they were taking. In fact, about two thirds of the patients with 'porphyria' in the clinic notes had other reasons, besides suspicion of AHP, for the presence of this word (**Table 9**). A large number of these patients were candidates for liver transplantation. Standard clinical documentation for evaluation for this procedure included a list of possible causes of liver failure, including protoporphyria. Porphyria was also mentioned as a precaution for certain medications or treatments given to some patients in this group, which included hydroxycholorquinone ferrous sulfate, therapeutic abortion, and UV light therapy for actinic keratosis.

Discussion

This work identified four likely and 18 possible patients who had no mention of porphyria in their charts for whom AHP diagnostic testing could be indicated. In addition, four patients who had mention of porphyria in their charts not related to a diagnostic evaluation of the disease were also found likely to have AHP diagnostic testing indicated. This number of patients with indications for AHP diagnostic testing and possibly to-be confirmed diagnosis vastly exceeds that due to chance and surpassed our expectations. It will require clinical follow-up to determine whether these patients' symptoms are truly due to AHP or not, but the manual record review clearly demonstrates that our methodology has found patients for whom a spot urine porphobilinogen test is indicated.

Another benefit of identifying such patients is to inform local specialists of the presence of patients with rare diseases in which they have expertise. An institution-wide search for confirmed AHP patients through our targeted ICD-10-CM code search plus manual chart review identified 30 confirmed AHP patients. A majority of these patients were previously unknown to

the porphyria specialist (TD) at OHSU. Identifying rare disease patients through large-scale data review in this manner can help connect them with the appropriate specialist to ensure optimal care.

Our results strongly suggest that leveraging of EHR data coupled with machine learning can be an effective method of identifying patients who should receive a diagnostic biochemical test to screen for AHP. Our automated model was able to identify patients with compelling constellations of symptoms who had not be previously worked up for porphyria. It was also able to identify patients for whom porphyria had been considered without direct access to porphyria-related data elements such as hemin treatment, lab tests specific to AHP, or mention of AHP diagnosis in clinical notes.

This is especially interesting in the light that the overall cross-validation scores of the model on the data set using the known 30 AHP cases as the positive set and the rest of the data as negative training samples was not very high, with cross-validation yielding an average AUC = 0.775. This is certainly a low performance figure compared to other current machine learning tasks such as publication type identification {Cohen, 2015 #9258}, or facial image recognition {Sun, 2015 #11641}. However, these other tasks are very different from this one due to the extremely rare nature of the positive AIP cases in both the training data as well as in the actual patient population. In most machine learning research, a data set is considered skewed or imbalanced if the number of positive cases is much less than 50%. A recent systematic review on imbalanced data classification cites articles investigating negative to positive case ratios of 100 to 1 as "highly imbalanced" {Kaur, 2019 #11902;Dhar, 2014 #11903}. For problems such as rare diseases, the imbalance ratio can be nearly 10,000 to 1, as it is here. Lifting the predictive power to perhaps 22 in 100 manually reviewed cases is a potentially transformative level of performance.

The strongest positive predictors in the model included unexplained abdominal pain, pelvic and perineal pain, nausea and vomiting, and a number of pain and nausea medications. Frequent urinalysis was also a strong positive predictive feature, this is likely due to being associated with frequent ER visits and hospitalizations. The model relied on encoding the frequency of episodes, and not just binary presence of absence of symptoms. Indirectly, in the model this represented recurrent, undiagnosed problems consistent with AHP.

As these methods are general, and not specific to AHP, they should be applicable to other rare disorders that have a constellation of recurrent symptoms as indicating features. There are likely ways to improve the machine learning approach, including the use of more advanced features that represent time, duration, and intervals, explicit coding of symptom separation and overlap, and more sophisticated machine learning algorithms specifically tailored to situations where the positive case is extremely rare. Investigation into machine learning algorithms for highly skewed data such as these is an active area of research {Haixiang, 2017 #11642}.

Conclusion

The combination of large data sets, machine learning techniques, and clinical knowledge engineering can be a powerful tool to identify patients with undiagnosed rare diseases. The use

case of AHP presented here revealed four undiagnosed patients thought likely to have AHP, as well as 18 others who would likely benefit from testing. This level of precision in identifying potential cases of AHP from EHR data is much higher than would be expected by the prevalence of the disease.

Analyzing the EHR with advanced techniques such as demonstrated here points to the potential of the future of digital medicine on a population scale. Advanced approaches enabled by the wide deployment of the EHR can now be used to improve medicine and medical care in areas that have been underserved or inaccessible. Health care can be made more proactive, not simply in terms of common conditions and age or gender related screening, but for rarer conditions as well.

We plan to continue this work in several directions. First, an IRB-approved clinical validation study is being implemented. In this study, we will contact the primary care clinicians (PCP) of the patients where AHP diagnostic testing was found to be *likely* or *possibly* indicated. We will inform them that an algorithm based on EHR data has determined that their patient might have AHP and could benefit from a spot urine porphobilinogen, which is an is inexpensive, non-invasive and easy to perform diagnostic test. With the agreement of the PCP, we will then contact patients and offer them the test. Expert clinical consultation will be made available to the PCP for any questions they have. We will collect data on the interactions with the PCPs, the number of spot urine porphobilinogen tests administered, as well as the test results. In this manner, we will be able to study the clinical impact of our rare disease identification approach.

Second, we will continue to refine our methods. Other machine learning algorithms, such as random forests and deep learning, may have advantages for AHP and other rare diseases. Other methods of encoding the EHR data that incorporate embeddings and temporal representations, have been shown to demonstrate leading-edge results in other fields, such as computer vision, machine translation, and speech recognition, and may assist with rare diseases.

Finally, we will extend this methodology to other rare diseases that are difficult to diagnose, focusing on those for which effective treatments are becoming available. If the timeline for diagnosing rate conditions can be substantially reduced, there is great potential to impact patient health in a very significant manner.

Acknowledgements and Funding

This work was funded and the associated editorial support was provided by Alnylam Pharmaceuticals, Inc., Cambridge, MA.

Declaration of Interest

Stephen Meninger, John J. Ko, and Jigar Amin, are employees of Alnylam, and Alex Wei was an employee of Alnylam during his contribution to the manuscript.

Table 1. Electronic Health Record (EHR) document types used in this research.

~~Administered Medications~~
~~Current Medications~~
~~Demographics~~
~~Encounter Diagnosis~~
~~Hospital Encounters~~
~~Lab Results~~
~~Medications Ordered~~
~~Microbiology Results~~
~~Notes~~
~~Problem List~~
~~Procedures Ordered~~
~~Lab Result Comments~~
~~Surgeries~~
~~Age~~

EHR Document Record Type	Description of Document
**Administered Medications**	Medications given to patient during a hiospital stay or ambulatory encounter.
**Current Medications**	The concomittent medications a patient is taking, as documented by providers during encounters.
**Demographics**	Patient demographic information
**Encounter Diagnosis**	The diagnoses and diagnostic codes assigned to a patient ambulatory encounter.
**Hospital Encounters**	Patient-level hospital admission information including times and billing codes.
**Lab Results**	Results of ordered lab tests including order time.
**Medications Ordered**	Medications ordered by for patients by clinicians during an encounter.
**Microbiology Results**	Results of microbiology lab tests in text form.
**Notes**	All types of clinical text including progress notes and discharge summaries.
**Problem List**	The concomittent list of active medical issues for a patient, as documented by providers during encounters.

Formatted: Font: 10 pt
Formatted: Font: 10 pt
Formatted: Font: 10 pt
Formatted: Font: 10 pt
Formatted: Font: 10 pt
Formatted: Font: 10 pt
Formatted: Font: 10 pt
Formatted: Font: 10 pt
Formatted: Font: 10 pt
Formatted: Font: 10 pt
Formatted: Font: 10 pt
Formatted: Font: 10 pt
Formatted: Font: 10 pt
Formatted: Font: 10 pt
Formatted Table
Formatted: Font: 12 pt
Formatted: Font: 12 pt
Formatted: Centered
Formatted: Font: 12 pt, Not Bold
Formatted ...
Formatted: Font: 12 pt, Not Bold
Formatted ...
Formatted: Font: 12 pt, Not Bold
Formatted ...
Formatted: Font: 12 pt, Not Bold
Formatted ...
Formatted: Font: 12 pt, Not Bold
Formatted ...
Formatted: Font: 12 pt, Not Bold
Formatted ...
Formatted: Font: 12 pt, Not Bold
Formatted ...
Formatted: Font: 12 pt, Not Bold
Formatted ...
Formatted: Font: 12 pt, Not Bold
Formatted ...
Formatted: Font: 12 pt, Not Bold
Formatted ...

**Procedures Ordered**	Procedures ordered by clinicians for patients during an encounter.
**Lab Result Comments**	Non-numerical, text portion, if any for results of lab tests.
**Surgeries**	Description of surgeries performed on patient at hospital in both text and coded forms.
Vitals	Documentation of vital values such as heartrate, blood pressure, weight, and temperature.

Formatted: Font: 12 pt, Not Bold

Formatted: Font: Not Bold

Formatted: Font: 12 pt, Not Bold

Formatted: Font: 12 pt, Not Bold

Formatted: Font: Not Bold

Formatted: Font: 12 pt, Not Bold

Formatted: Font: 12 pt, Not Bold

Formatted: Font: Not Bold

Formatted: Font: 12 pt, Not Bold

Formatted: Font: 12 pt, Not Bold

Formatted: Font: Not Bold

Formatted: Font: 12 pt, Not Bold

Table 2. Electronic Health Record (EHR) total document and unique patients counts of porphyria codes and mentioned in text notes or label tests. Counts shown here are out of a total of 347,709,284 individual EHR documents and 204, 413 total unique patient records.

**Code**	~~**Total**~~**Total Documents**	**Patients**
ICD9 277.1	3879	308
E80.0 Hereditary erythropoietic porphyria	472	37
E80.1 Porphyria cutanea tarda	783	77
E80.20 Unspecified porphyria	2010	247
E80.21 Acute intermittent (hepatic) porphyria	1016	47
E80.29 Other porphyria	109	24
E80.4 Gilbert syndrome	3197	366
E80.6 Other disorders of bilirubin metabolism	9502	2308
E80.7 Disorder of bilirubin metabolism, unspecified	75	58
Patients with porphyria mentioned in a lab test:	359	175
Searching field NOTE_TEXT for term porphyria:	14353	3012

Formatted: Normal

Formatted: Font: (Default) Times New Roman

Formatted: Font: (Default) Times New Roman

Formatted: Font: (Default) Times New Roman

Formatted: Font: (Default) Calibri, Font color: Black

Formatted Table

Formatted: Font color: Text 1

Table 3. Summary of document types and counts used in the EHR data set for this research.

Document Type	Patients	Encounters	Records	Median	Max
Current Medications	187724	N/A	99602443	89	57406
Demographics	204413	N/A	204413	1	1
Encounter Attributes	204412	19589057	19589057	43	3335
Encounter Diagnoses	202843	10113657	52295188	69	27215
Hospital Encounters	145551	1163284	1163284	3	520
Lab Results	172795	2012185	58386934	84	27384
Ordered Medications	190256	3964120	15155203	23	7041
Microbiology Results	54798	145528	1988429	5	5174
Notes	204161	10014987	28938900	56	14933
Problem List	181221	N/A	1737749	6	204
Procedures Ordered	198833	5129756	19501225	31	35364
Result Comments	131104	896896	1542279	4	1765
Surgeries	44238	78403	83535	1	54
Vitals	199971	3500418	18268032	24	9442
Administered Medications	100565	349332	17160858	17	53178
Ambulatory Encounters	204235	12091755	12091755	27	1991

Type	Patients	Encounters	Records	Mean	Median	Max
current_medications	187,724	N/A	99,602,443	530.58	89	57,406
demographics	204,413	N/A	204,413	1.00	1	1
encounter_attributes	204412	19,589,057	19,589,057	95.83	43	3335
encounter_diagnoses	202,843	10,113,657	52,295,188	257.81	69	27,215
hospital_encounters	145,551	1,163,284	1,163,284	7.99	3	520
lab_results	172,795	2,012,185	58,386,934	337.90	84	27,384
medications_ordered	190,256	3,964,120	15,155,203	79.66	23	7,041
microbiology_results	54,798	145,528	1,988,429	36.29	5	5,174
notes	204,161	10,014,987	28,938,900	141.75	56	14,933
problem_list	181,221	N/A	1,737,749	9.59	6	204
procedures_ordered	198,833	5,129,756	19,501,225	98.08	31	35,364
result_comments	131,104	896,896	1,542,279	11.76	4	1,765
surgeries	44,238	78,403	83,535	1.89	1	54
vitals	199,971	3,500,418	18,268,032	91.35	24	9,442
administered_medications	100,565	349,332	17,160,858	170.64	17	53,178
ambulatory_encounters	204,235	12,091,755	12,091,755	59.21	27	1,991

Commented [AMC10]: Bill, What do you think of ...

Table 4. Cross-validation performance of the final feature set on the entire data set for ranking the 30 confirmed cases of porphyria higher than the general population. SVM with radial basis function (RBF) kernel and gamma = 0.04.

**Metric**	**Score**
**AUC**	0.775
**Average Precision**	0.060
**Precision @ 100**	0.031
**Log Loss**	0.404

Formatted: Font: (Default) Times New Roman, Font color: Text 1

Formatted Table

Formatted: Font: (Default) Times New Roman

Formatted: Font: (Default) Times New Roman

Formatted: Font: (Default) Times New Roman

Formatted: Font: (Default) Times New Roman

Table 5[illegible]sessment of the likelihood of undiagnosed acute hepatic porphyria based on clinical note symptom documentation. Both groups of 100 reviewed patients are listed.

	Acute Hepatic Porphyria?	# Patients
No mention of porphyria group (n=100)	Diagnostic test is *Likely Indicated*	4
	Diagnostic test is *Possibly Indicated*	18
	Diagnostic test is *Unlikely Indicated*	68
	Deceased	10
'Porph' in clinical notes group (n=100)	Suspected in chart	16
	Suspected, ruled out in chart	15
	Diagnostic test is *Possibly Indicated*, not suspected in chart	4
	Unlikely based on chart review	54
	Diagnosed, documented in chart	4
	Unknown, unable to determine	1
	Deceased	6

Formatted Table

Table 6. Top alternative explanations for AHP symptom profiles seen in both group[illegible]atients. Conditions seen in no more than one patient are not listed.

	**Alternate AHP Symptom Explanation**	**# Patients**
No mention of porphyria group	Surgery	8
	Inflammatory Bowel Disease	6
	Cancer	6
	Cancer Chemotherapy	5
	Gallbladder Pathology	4
	Diabetes	3
	Carnitine Palmitoyl Transferase Deficiency	2
	Renal	4
	Poly Cystic Ovarian Syndrome	2
	Appendicitis	2
	Mastocytosis	2
'Porph' in clinical notes group	Liver Pathology	30
	Chemotherapy/Drug Side Effects	3
	Mastocytosis	2

Formatted Table

Table 7. Age statistics in years for the two patient groups.

	NO MENTION OF PORPHYRIA	'PORPH' IN CLINICAL NOTES
**MEDIAN**	51	54
**MEAN**	53	50
**MIN**	8	6
**MAX**	91	91

Formatted Table

Table 8. Sex distribution for the two patient groups.

	NO MENTION OF PORPHYRIA	'POPRH' IN CLINICAL NOTES
**MALE**	25	44
**FEMALE**	75	56

Formatted Table

Table 9. Top reasons for the presence of the word 'porph' found in the clinical note.

More Common Reasons for 'Porph' in Clinical Notes	**# Patients**
Suspicion of Porphyria	31
Liver Transplant Documentation	30
Porphyria Mentioned in Treatment Precautions	18
Porphyria Diagnosis Mentioned in Notes	4
Porphyria Lab Tests Listed for Screening Physical	3
Family History of Porphyria	5
Misspelling	2

Formatted Table

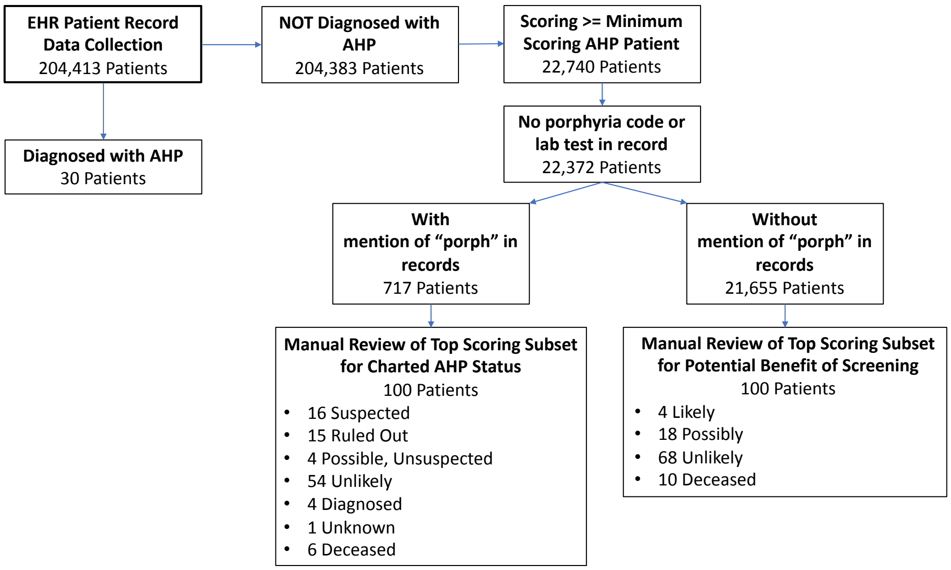

Figure 1. Flowchart of patient data record selection. Collection starts from full set of from full collection 204, 413 patient records and is filtered down to two sets of 100 records that were manually reviewed and characterized for 1) present indications for screening for AHP, and 2) status of AHP evaluation in the clinical notes of the record.

References

Supplemental Table 1. Final 141~~6~~ features selected for inclusion in the machine learning model to predict acute hepatic porphyria. Features are scored by number of occurrances in an individual patient medical record, and then normalized.

INDEX	FEATURE	SOURCE DOCUMENTS	DES[illegible]
1	ABDOMINAL_PAIN_DX_NAME	Encounter Diagnosis, Patient Problem List	Tex[illegible] of d[illegible] code[illegible]
2	ABDOMINAL_PAIN_UNSPECIFIED_SITE_DX_NAME	Encounter Diagnosis, Patient Problem List	Tex[illegible] of diagnosis cod[illegible]
3	ALTERNATIVE_THERAPY_-_PINEAL_HORMONE_AGENTS_PHARM_SUBCLASS_NAME	Concomittent Medications, Administered Medications, Medications Ordered	Text description of d[illegible]
4	ANALGESIC_OPIOID_OXYCODONE_COMBINATIONS_PHARM_SUBCLASS_NAME	Concomittent Medications, Administered Medications, Medications Ordered	Text description of d[illegible]
5	ANTI-ANXIETY_-_BENZODIAZEPINES_PHARM_CLASS_NAME	Concomittent Medications, Administered Medications, Medications Ordered	Text description of d[illegible]
6	ANTICONVULSANT_-_GABA_ANALOGS_PHARM_SUBCLASS_NAME	Concomittent Medications, Administered Medications, Medications Ordered	Text description of d[illegible]
7	ANTIEMETIC_-_PHENOTHIAZINES_PHARM_SUBCLASS_NAME	Concomittent Medications, Administered Medications, Medications Ordered	Text description of d[illegible]
8	ANTIHISTAMINE_-_1ST_GENERATION_-_ETHANOLAMINES_PHARM_SUBCLASS_NAME	Concomittent Medications, Administered Medications, Medications Ordered	Text description of d[illegible]
9	ANTIHISTAMINE_-_1ST_GENERATION_-_PHENOTHIAZINES_PHARM_SUBCLASS_NAME	Concomittent Medications, Administered Medications, Medications Ordered	Text description of d[illegible]
10	BASO_#_COMPONENT_NAME	Lab Results	Percent Basophils perf[illegible]

Commented [AMC12]: Need to reformat table to include data descriptions

Formatted: Font: (Default) Times New Roman, 9 pt

Formatted: Font: Bold

Formatted: Font: (Default) Times New Roman, 9 pt

Formatted Table

Formatted: Font: (Default) Times New Roman, 9 pt

Formatted: Font: (Default) Times New Roman, 9 pt

Formatted: Font: (Default) Times New Roman, 9 pt

Formatted: Font: (Default) Times New Roman, 9 pt

Formatted: Font: (Default) Times New Roman, 9 pt

Formatted: Font: (Default) Times New Roman, 9 pt

Formatted: Font: (Default) Times New Roman, 9 pt

Formatted: Font: (Default) Times New Roman, 9 pt

Formatted: Font: (Default) Times New Roman, 9 pt

Formatted: Font: (Default) Times New Roman, 9 pt

11	CALCIUM_REPLACEMENT_PHARM_CLASS_NAME	Concomittent Medications, Administered Medications, Medications Ordered	Text description of d[illegible]	**Formatted:** Font: (Default) Times New Roman, 9 pt
12	CBC_WITH_DIFFERENTIAL_PROC_NAME	Procedures Ordered	CBC with diff orde[illegible]	**Formatted:** Font: (Default) Times New Roman, 9 pt
13	CNSLT0031_PROC_CODE	Procedures Ordered	Code for consult to Gas[illegible]	**Formatted:** Font: (Default) Times New Roman, 9 pt
14	CONSULT_TO_GASTROENTEROLOGY_PROC_NAME	Procedures Ordered	Consult to Gastoenterology orde[illegible]	**Formatted:** Font: (Default) Times New Roman, 9 pt
15	COPD_(CHRONIC_OBSTRUCTIVE_PULMONARY_DISEASE)_(HCC)_DX_NAME	Encounter Diagnosis, Patient Problem List	Text description of diagnosis cod[illegible]	**Formatted:** Font: (Default) Times New Roman, 9 pt
16	CREATININE_URINE_CONCENTRATION_COMPONENT_NAME	Lab Results	lab result component pres[illegible]	**Formatted:** Font: (Default) Times New Roman, 9 pt
17	CREATININEUR(REFERRAL)_COMPONENT_NAME	Lab Results	lab result component pres[illegible]	**Formatted:** Font: (Default) Times New Roman, 9 pt
18	DIFFERENTIAL_PROC_NAME	Procedures Ordered	blood differential order pres[illegible]	**Formatted:** Font: (Default) Times New Roman, 9 pt
19	DIPHENHYDRAMINE_HCL_GENERIC_NAME_1	Concomittent Medication, Medications Ordered	Generic name of med[illegible]	**Formatted:** Font: (Default) Times New Roman, 9 pt
20	ELEVATED_WHITE_BLOOD_CELL_COUNT_UNSPECIFIED_DX_ICD10_NAME	Encounter Diagnosis, Patient Problem List	Text description of diagnosis cod[illegible]	**Formatted:** Font: (Default) Times New Roman, 9 pt
21	EOS_#_COMPONENT_NAME	Lab Results	eosinaphil count lab [illegible]	**Formatted:** Font: (Default) Times New Roman, 9 pt
22	ESSENTIAL_(PRIMARY)_HYPERTENSION_DX_ICD10_NAME	Encounter Diagnosis, Patient Problem List	Text description of diagnosis cod[illegible]	**Formatted:** Font: (Default) Times New Roman, 9 pt
23	FERRITIN_SERUM_PROC_NAME	Procedures Ordered	serum ferritin orde[illegible]	**Formatted:** Font: (Default) Times New Roman, 9 pt
24	HYDROMORPHONE_HCL_GENERIC_NAME_1	Concomittent Medication, Medications Ordered	Generic name of med[illegible]	**Formatted:** Font: (Default) Times New Roman, 9 pt
25	LAB00047_PROC_CODE	Procedures Ordered	Plasma lipase procedure orde[illegible]	**Formatted:** Font: (Default) Times New Roman, 9 pt
26	LAB00364_PROC_CODE	Procedures Ordered	Microscopic urine exam orde[illegible]	**Formatted:** Font: (Default) Times New Roman, 9 pt
27	LAB00681_PROC_CODE	Procedures Ordered	CBC with differential orde[illegible]	**Formatted:** Font: (Default) Times New Roman, 9 pt

28	LAB100107_PROC_CODE	Procedures Ordered	Blood differential orde	**Formatted:** Font: (Default) Times New Roman, 9 pt
29	LAB100227_PROC_CODE	Procedures Ordered	Urine volume measurement orde	**Formatted:** Font: (Default) Times New Roman, 9 pt
30	LAB100882_PROC_CODE	Procedures Ordered	Multi-tube blood drav	**Formatted:** Font: (Default) Times New Roman, 9 pt
31	LIPASE__(LAB)_COMPONENT_NAME	Lab Results	plasma lipase result component pres	**Formatted:** Font: (Default) Times New Roman, 9 pt
32	LIPASE_PLASMA_PROC_NAME	Procedures Ordered	plasma lipase orde	**Formatted:** Font: (Default) Times New Roman, 9 pt
33	LYMPHOCYTE_#_COMPONENT_NAME	Lab Results	blood lymphocyte count results pres	**Formatted:** Font: (Default) Times New Roman, 9 pt
34	MAGNESIUM_SALTS_REPLACEMENT_PHARM_CLASS_NAME	Concomittent Medications, Administered Medications, Medications Ordered	Text description of d	**Formatted:** Font: (Default) Times New Roman, 9 pt
35	MELATONIN_GENERIC_NAME_1	Concomittent Medication, Medications Ordered	Generic name of med	**Formatted:** Font: (Default) Times New Roman, 9 pt
36	MINERALS_AND_ELECTROLYTES_-_CALCIUM_REPLACEMENT/VITAMIN_D_COMBINATIONS_PHARM_SUBCLASS_NAME	Concomittent Medications, Administered Medications, Medications Ordered	Text description of d	**Formatted:** Font: (Default) Times New Roman, 9 pt
37	MISC_REF_TEST_NAME_COMPONENT_NAME	Lab Results	Special test given with name of test in RES	**Formatted:** Font: (Default) Times New Roman, 9 pt
38	MISC_REF_TEST_RESULT_COMPONENT_NAME	Lab Results	Result of special test	**Formatted:** Font: (Default) Times New Roman, 9 pt
39	MONOCYTE_#_COMPONENT_NAME	Lab Results	blood monocyte count results pres	**Formatted:** Font: (Default) Times New Roman, 9 pt
40	NAUSEA_WITH_VOMITING_UNSPECIFIED_DX_ICD10_NAME	Encounter Diagnosis, Patient Problem List	Text description of diagnosis cod	**Formatted:** Font: (Default) Times New Roman, 9 pt
41	NEUTROPHIL_#_COMPONENT_NAME	Lab Results	blood neutrophil count results pres	**Formatted:** Font: (Default) Times New Roman, 9 pt
42	NGRAM_0^pramipexole	Notes	Bigram of [token]^[token] found in free text	**Formatted:** Font: (Default) Times New Roman, 9 pt
43	NGRAM_0^tablet	Notes	Bigram of [token]^[token] found in free text	**Formatted:** Font: (Default) Times New Roman, 9 pt
44	NGRAM_10^olanzapine	Notes	Bigram of [tok	**Formatted:** Font: (Default) Times New Roman, 9 pt

			found in free text.
45	NGRAM_10^tablet	Notes	Bigram of [token]^[token] found in free text
46	NGRAM_100^sodium	Notes	Bigram of [token]^[token] found in free text
47	NGRAM_4^mg	Notes	Bigram of [token]^[token] found in free text
48	NGRAM_4^odt	Notes	Bigram of [token]^[token] found in free text
49	NGRAM_90^albuterol	Notes	Bigram of [token]^[token] found in free text
50	NGRAM_abdominal	Notes	Unigram of [token] found in free
51	NGRAM_abdominal^pain	Notes	Bigram of [token]^[token] found in free text
52	NGRAM_acute	Notes	Unigram of [token] found in free
53	NGRAM_acute^distress	Notes	Bigram of [token]^[token] found in free text
54	NGRAM_ambulatory	Notes	Unigram of [token] found in free
55	NGRAM_antibiotics	Notes	Unigram of [token] found in free
56	NGRAM_antibiotics^sulfonamide	Notes	Bigram of [token]^[token] found in free text
57	NGRAM_atraumatic	Notes	Unigram of [token] found in free
58	NGRAM_bipolar	Notes	Unigram of [token] found in free
59	NGRAM_cigarettes	Notes	Unigram of [token] found in free
60	NGRAM_compazine	Notes	Unigram of [token] found in free
61	NGRAM_control^pain	Notes	Bigram of [tok[illegible]

Formatted: Font: (Default) Times New Roman, 9 pt
Formatted: Font: (Default) Times New Roman, 9 pt
Formatted: Font: (Default) Times New Roman, 9 pt
Formatted: Font: (Default) Times New Roman, 9 pt
Formatted: Font: (Default) Times New Roman, 9 pt
Formatted: Font: (Default) Times New Roman, 9 pt
Formatted: Font: (Default) Times New Roman, 9 pt
Formatted: Font: (Default) Times New Roman, 9 pt
Formatted: Font: (Default) Times New Roman, 9 pt
Formatted: Font: (Default) Times New Roman, 9 pt
Formatted: Font: (Default) Times New Roman, 9 pt
Formatted: Font: (Default) Times New Roman, 9 pt
Formatted: Font: (Default) Times New Roman, 9 pt
Formatted: Font: (Default) Times New Roman, 9 pt
Formatted: Font: (Default) Times New Roman, 9 pt
Formatted: Font: (Default) Times New Roman, 9 pt
Formatted: Font: (Default) Times New Roman, 9 pt
Formatted: Font: (Default) Times New Roman, 9 pt

			found in free text.
62	NGRAM_depakote	Notes	Unigram of [token] found in free
63	NGRAM_dilaudid	Notes	Unigram of [token] found in free
64	NGRAM_discharged	Notes	Unigram of [token] found in free
65	NGRAM_disintegrating	Notes	Unigram of [token] found in free
66	NGRAM_docusate	Notes	Unigram of [token] found in free
67	NGRAM_docusate^sodium	Notes	Bigram of [token]^[token] found in free text
68	NGRAM_dose^oral	Notes	Bigram of [token]^[token] found in free text
69	NGRAM_duloxetine	Notes	Unigram of [token] found in free
70	NGRAM_ed	Notes	Unigram of [token] found in free
71	NGRAM_edisylate]	Notes	Unigram of [token] found in free
72	NGRAM_extended^tablet	Notes	Bigram of [token]^[token] found in free text
73	NGRAM_fibromyalgia	Notes	Unigram of [token] found in free
74	NGRAM_flare	Notes	Unigram of [token] found in free
75	NGRAM_flares	Notes	Unigram of [token] found in free
76	NGRAM_focal	Notes	Unigram of [token] found in free
77	NGRAM_gallops	Notes	Unigram of [token] found in free
78	NGRAM_genitourinary	Notes	Unigram of [token] found in free
79	NGRAM_glycol	Notes	Unigram of [token] found in free
80	NGRAM_glycol^polyethylene	Notes	Bigram of [tok

Formatted: Font: (Default) Times New Roman, 9 pt

Formatted: Font: (Default) Times New Roman, 9 pt

Formatted: Font: (Default) Times New Roman, 9 pt

Formatted: Font: (Default) Times New Roman, 9 pt

Formatted: Font: (Default) Times New Roman, 9 pt

Formatted: Font: (Default) Times New Roman, 9 pt

Formatted: Font: (Default) Times New Roman, 9 pt

Formatted: Font: (Default) Times New Roman, 9 pt

Formatted: Font: (Default) Times New Roman, 9 pt

Formatted: Font: (Default) Times New Roman, 9 pt

Formatted: Font: (Default) Times New Roman, 9 pt

Formatted: Font: (Default) Times New Roman, 9 pt

Formatted: Font: (Default) Times New Roman, 9 pt

Formatted: Font: (Default) Times New Roman, 9 pt

Formatted: Font: (Default) Times New Roman, 9 pt

Formatted: Font: (Default) Times New Roman, 9 pt

Formatted: Font: (Default) Times New Roman, 9 pt

Formatted: Font: (Default) Times New Roman, 9 pt

Formatted: Font: (Default) Times New Roman, 9 pt

			found in free text.	
81	NGRAM_gram	Notes	Unigram of [token] found in free	**Formatted:** Font: (Default) Times New Roman, 9 pt
82	NGRAM_hydromorphone	Notes	Unigram of [token] found in free	**Formatted:** Font: (Default) Times New Roman, 9 pt
83	NGRAM_instructed	Notes	Unigram of [token] found in free	**Formatted:** Font: (Default) Times New Roman, 9 pt
84	NGRAM_iv	Notes	Unigram of [token] found in free	**Formatted:** Font: (Default) Times New Roman, 9 pt
85	NGRAM_latex	Notes	Unigram of [token] found in free	**Formatted:** Font: (Default) Times New Roman, 9 pt
86	NGRAM_magnesium	Notes	Unigram of [token] found in free	**Formatted:** Font: (Default) Times New Roman, 9 pt
87	NGRAM_melatonin	Notes	Unigram of [token] found in free	**Formatted:** Font: (Default) Times New Roman, 9 pt
88	NGRAM_miralax	Notes	Unigram of [token] found in free	**Formatted:** Font: (Default) Times New Roman, 9 pt
89	NGRAM_mouth^needed	Notes	Bigram of [token]^[token] found in free text	**Formatted:** Font: (Default) Times New Roman, 9 pt
90	NGRAM_mouth^twelve	Notes	Bigram of [token]^[token] found in free text	**Formatted:** Font: (Default) Times New Roman, 9 pt
91	NGRAM_nausea	Notes	Unigram of [token] found in free	**Formatted:** Font: (Default) Times New Roman, 9 pt
92	NGRAM_nausea^vomiting	Notes	Bigram of [token]^[token] found in free text	**Formatted:** Font: (Default) Times New Roman, 9 pt
93	NGRAM_odt	Notes	Unigram of [token] found in free	**Formatted:** Font: (Default) Times New Roman, 9 pt
94	NGRAM_odt^ondansetron	Notes	Bigram of [token]^[token] found in free text	**Formatted:** Font: (Default) Times New Roman, 9 pt
95	NGRAM_olanzapine	Notes	Unigram of [token] found in free	**Formatted:** Font: (Default) Times New Roman, 9 pt
96	NGRAM_oncology	Notes	Unigram of [token] found in free	**Formatted:** Font: (Default) Times New Roman, 9 pt
97	NGRAM_ondansetron	Notes	Unigram of [token] found in free	**Formatted:** Font: (Default) Times New Roman, 9 pt
98	NGRAM_oral^powder	Notes	Bigram of [token]^[token] found in free text	**Formatted:** Font: (Default) Times New Roman, 9 pt

99	NGRAM_oxycodone	Notes	Unigram of [token] found in free
100	NGRAM_pain^severe	Notes	Bigram of [token]^[token] found in free text
101	NGRAM_pathology	Notes	Unigram of [token] found in free
102	NGRAM_penicillins	Notes	Unigram of [token] found in free
103	NGRAM_phenergan	Notes	Unigram of [token] found in free
104	NGRAM_polyethylene	Notes	Unigram of [token] found in free
105	NGRAM_powder	Notes	Unigram of [token] found in free
106	NGRAM_pramipexole	Notes	Unigram of [token] found in free
107	NGRAM_propranolol	Notes	Unigram of [token] found in free
108	NGRAM_protocol	Notes	Unigram of [token] found in free
109	NGRAM_psychosis	Notes	Unigram of [token] found in free
110	NGRAM_risperidone	Notes	Unigram of [token] found in free
111	NGRAM_rubs	Notes	Unigram of [token] found in free
112	NGRAM_scoliosis	Notes	Unigram of [token] found in free
113	NGRAM_seroquel	Notes	Unigram of [token] found in free
114	NGRAM_severe	Notes	Unigram of [token] found in free
115	NGRAM_stomach	Notes	Unigram of [token] found in free
116	NGRAM_sulfa	Notes	Unigram of [token] found in free
117	NGRAM_sulfonamide	Notes	Unigram of [token] found in free
118	NGRAM_urine	Notes	Unigram of [token] found in free

Formatted: Font: (Default) Times New Roman, 9 pt

Formatted: Font: (Default) Times New Roman, 9 pt
Formatted: Font: (Default) Times New Roman, 9 pt
Formatted: Font: (Default) Times New Roman, 9 pt
Formatted: Font: (Default) Times New Roman, 9 pt
Formatted: Font: (Default) Times New Roman, 9 pt
Formatted: Font: (Default) Times New Roman, 9 pt
Formatted: Font: (Default) Times New Roman, 9 pt
Formatted: Font: (Default) Times New Roman, 9 pt
Formatted: Font: (Default) Times New Roman, 9 pt
Formatted: Font: (Default) Times New Roman, 9 pt
Formatted: Font: (Default) Times New Roman, 9 pt
Formatted: Font: (Default) Times New Roman, 9 pt
Formatted: Font: (Default) Times New Roman, 9 pt
Formatted: Font: (Default) Times New Roman, 9 pt
Formatted: Font: (Default) Times New Roman, 9 pt
Formatted: Font: (Default) Times New Roman, 9 pt
Formatted: Font: (Default) Times New Roman, 9 pt
Formatted: Font: (Default) Times New Roman, 9 pt
Formatted: Font: (Default) Times New Roman, 9 pt

119	NGRAM_vicodin	Notes	Unigram of [token] found in free [illegible]	**Formatted:** Font: (Default) Times New Roman, 9 pt
120	NGRAM_zofran	Notes	Unigram of [token] found in free [illegible]	**Formatted:** Font: (Default) Times New Roman, 9 pt
121	NORMAL_RANGE_COMPONENT_NAME	Lab Results	Lab test result within normal rang[illegible]	**Formatted:** Font: (Default) Times New Roman, 9 pt
122	OBSTRUCTIVE_SLEEP_APNEA_(ADULT)_(PEDIATRIC)_DX_ICD10_NAME	Encounter Diagnosis, Patient Problem List	Text description of diagnosis cod[illegible]	**Formatted:** Font: (Default) Times New Roman, 9 pt
123	OBSTRUCTIVE_SLEEP_APNEA_DX_NAME	Encounter Diagnosis, Patient Problem List	Text description of diagnosis cod[illegible]	**Formatted:** Font: (Default) Times New Roman, 9 pt
124	ONDANSETRON_HCL_GENERIC_NAME_1	Concomittent Medication, Medications Ordered	Generic name of med[illegible]	**Formatted:** Font: (Default) Times New Roman, 9 pt
125	OXYCODONE_HCL/ACETAMINOPHEN_GENERIC_NAME_1	Concomittent Medication, Medications Ordered	Generic name of med[illegible]	**Formatted:** Font: (Default) Times New Roman, 9 pt
126	PATHOLOGY_PROC_NAME	Procedures Ordered	Transcribed pathology report pres[illegible]	**Formatted:** Font: (Default) Times New Roman, 9 pt
127	PELVIC_AND_PERINEAL_PAIN_DX_ICD10_NAME	Encounter Diagnosis, Patient Problem List	Text description of diagnosis cod[illegible]	**Formatted:** Font: (Default) Times New Roman, 9 pt
128	PINEAL_HORMONE_AGENTS_PHARM_CLASS_NAME	Concomittent Medications, Administered Medications, Medications Ordered	Text description of d[illegible]	**Formatted:** Font: (Default) Times New Roman, 9 pt
129	PROCHLORPERAZINE_EDISYLATE_GENERIC_NAME_1	Concomittent Medication, Medications Ordered	Generic name of med[illegible]	**Formatted:** Font: (Default) Times New Roman, 9 pt
130	PROMETHAZINE_HCL_GENERIC_NAME_1	Concomittent Medication, Medications Ordered	Generic name of med[illegible]	**Formatted:** Font: (Default) Times New Roman, 9 pt
131	RADIOLOGY_PROC_NAME	Procedures Ordered	Transcribed radiology report pres[illegible]	**Formatted:** Font: (Default) Times New Roman, 9 pt
132	RAINBOW_HOLD_TUBE_-_BLUE_TOP_PROC_NAME	Procedures Ordered	Multi-tube blood draw[illegible]	**Formatted:** Font: (Default) Times New Roman, 9 pt
133	RESTLESS_LEGS_SYNDROME_DX_ICD10_NAME	Encounter Diagnosis, Patient Problem List	Text description of diagnosis cod[illegible]	**Formatted:** Font: (Default) Times New Roman, 9 pt
134	TOBACCO_ABUSE_DX_NAME	Encounter Diagnosis, Patient Problem List	Text description of diagnosis cod[illegible]	**Formatted:** Font: (Default) Times New Roman, 9 pt

135	TRIPLE_P04_CRYSTALS_COMPONENT_NAME	Lab Results	Component of resu[illegible]
136	TRNS00039_PROC_CODE	Procedures Ordered	Transcribed pathology report pres[illegible]
137	TRNS00040_PROC_CODE	Procedures Ordered	Transcribed imaging report pres[illegible]
138	UNSPECIFIED_ABDOMINAL_PAIN_DX_ICD10_NAME	Encounter Diagnosis, Patient Problem List	Text description of diagnosis cod[illegible]
139	UNSPECIFIED_ABDOMINAL_PAIN_DX_ICD10_NAME	Encounter Diagnosis, Patient Problem List	Text description of diagnosis cod[illegible]
140	URINE_MICROSCOPIC_EXAM_PROC_NAME	Lab Results	Name of lab test proc[illegible]
141	VOL(URINE)_PROC_NAME	Lab Results	Name of lab test proc[illegible]

Formatted: Font: (Default) Times New Roman, 9 pt
Formatted: Font: (Default) Times New Roman, 9 pt
Formatted: Font: (Default) Times New Roman, 9 pt
Formatted: Font: (Default) Times New Roman, 9 pt
Formatted: Font: (Default) Times New Roman, 9 pt
Formatted: Font: (Default) Times New Roman, 9 pt
Formatted: Font: (Default) Times New Roman, 9 pt

1. PELVIC_AND_PERINEAL_PAIN_DX_ICD10_NAME
2. MAGNESIUM_SALTS_REPLACEMENT_PHARM_CLASS_NAME
3. NGRAM_atraumatic
4. NGRAM_pain^severe
5. NAUSEA_WITH_VOMITING_UNSPECIFIED_DX_ICD10_NAME
6. CALCIUM_REPLACEMENT_PHARM_CLASS_NAME
7. MINERALS_AND_ELECTROLYTES_-_CALCIUM_REPLACEMENT/VITAMIN_D_COMBINATIONS_PHARM_SUBCLASS_NAME
8. NGRAM_compazine
9. DIFFERENTIAL_PROC_NAME
10. LAB100107_PROC_CODE
11. COPD_(CHRONIC_OBSTRUCTIVE_PULMONARY_DISEASE)_(HCC)_DX_NAME
12. ELEVATED_WHITE_BLOOD_CELL_COUNT_UNSPECIFIED_DX_ICD10_NAME
13. OBSTRUCTIVE_SLEEP_APNEA_(ADULT)_(PEDIATRIC)_DX_ICD10_NAME
14. NGRAM_oxycodone
15. NGRAM_dose^oral
16. PROCHLORPERAZINE_EDISYLATE_GENERIC_NAME_1
17. NGRAM_protocol
18. NGRAM_scoliosis
19. NGRAM_duloxetine
20. ANTIEMETIC_-_PHENOTHIAZINES_PHARM_SUBCLASS_NAME
21. NGRAM_seroquel
22. TOBACCO_ABUSE_DX_NAME
23. HYDROMORPHONE_HCL_GENERIC_NAME_1
24. OBSTRUCTIVE_SLEEP_APNEA_DX_NAME
25. NGRAM_oncology
26. LAB100882_PROC_CODE

27. RAINBOW_HOLD_TUBE_-_BLUE_TOP_PROC_NAME
28. NGRAM_mouth^twelve
29. DIPHENHYDRAMINE_HCL_GENERIC_NAME_1
30. NGRAM_extended^tablet
31. ANTIHISTAMINE_-_1ST_GENERATION_-_ETHANOLAMINES_PHARM_SUBCLASS_NAME
32. NGRAM_cigarettes
33. UNSPECIFIED_ABDOMINAL_PAIN_DX_ICD10_NAME
34. NGRAM_fibromyalgia
35. NGRAM_bipolar
36. # REMOVED NGRAM_hematology
37. LAB00364_PROC_CODE
38. URINE_MICROSCOPIC_EXAM_PROC_NAME
39. NGRAM_edisylate]
40. ANTI-ANXIETY_-_BENZODIAZEPINES_PHARM_CLASS_NAME
41. ALTERNATIVE_THERAPY_-_PINEAL_HORMONE_AGENTS_PHARM_SUBCLASS_NAME
42. NGRAM_4^mg
43. ONDANSETRON_HCL_GENERIC_NAME_1
44. TRNS00039_PROC_CODE
45. PATHOLOGY_PROC_NAME
46. UNSPECIFIED_ABDOMINAL_PAIN_DX_ICD10_NAME
47. RESTLESS_LEGS_SYNDROME_DX_ICD10_NAME
48. TRNS00040_PROC_CODE
49. RADIOLOGY_PROC_NAME
50. NGRAM_miralax
51. CONSULT_TO_GASTROENTEROLOGY_PROC_NAME
52. CNSLT0031_PROC_CODE
53. NGRAM_ondansetron
54. ABDOMINAL_PAIN_DX_NAME
55. MELATONIN_GENERIC_NAME_1
56. PINEAL_HORMONE_AGENTS_PHARM_CLASS_NAME
57. TRIPLE_P04_CRYSTALS_COMPONENT_NAME
58. NGRAM_dilaudid
59. NGRAM_focal
60. NGRAM_nausea^vomiting
61. NGRAM_10^olanzapine
62. NGRAM_antibiotics
63. LAB00047_PROC_CODE
64. LIPASE_PLASMA_PROC_NAME
65. NGRAM_instructed
66. LIPASE__(LAB)_COMPONENT_NAME
67. NGRAM_4^odt
68. NGRAM_100^sodium
69. VOL(URINE)_PROC_NAME
70. LAB100227_PROC_CODE

71. NEUTROPHIL_#_COMPONENT_NAME
72. LYMPHOCYTE_#_COMPONENT_NAME
73. MONOCYTE_#_COMPONENT_NAME
74. EOS_#_COMPONENT_NAME
75. BASO_#_COMPONENT_NAME
76. NGRAM_10^tablet
77. OXYCODONE_HCL/ACETAMINOPHEN_GENERIC_NAME_1
78. NGRAM_olanzapine
79. NGRAM_genitourinary
80. ANALGESIC_OPIOID_OXYCODONE_COMBINATIONS_PHARM_SUBCLASS_NAME
81. NGRAM_90^albuterol
82. NGRAM_disintegrating
83. ANTICONVULSANT_-_GABA_ANALOGS_PHARM_SUBCLASS_NAME
84. NGRAM_risperidone
85. NGRAM_0^pramipexole
86. NORMAL_RANGE_COMPONENT_NAME
87. # REMOVED HISTAMINE_H2-RECEPTOR_INHIBITORS_PHARM_CLASS_NAME
88. # REMOVED GASTRIC_ACID_SECRETION_REDUCERS_-_HISTAMINE_H2-RECEPTOR_ANTAGONISTS_PHARM_SUBCLASS_NAME
89. NGRAM_abdominal
90. NGRAM_0^tablet
91. NGRAM_pramipexole
92. # REMOVED NGRAM_17^gram
93. ABDOMINAL_PAIN_UNSPECIFIED_SITE_DX_NAME
94. NGRAM_propranolol
95. NGRAM_rubs
96. # REMOVED NGRAM_infusion
97. NGRAM_pathology
98. NGRAM_control^pain
99. NGRAM_flare
100. NGRAM_hydromorphone
101. CREATININE_URINE_CONCENTRATION_COMPONENT_NAME
102. NGRAM_acute^distress
103. NGRAM_sulfonamide
104. NGRAM_antibiotics^sulfonamide
105. NGRAM_depakote
106. NGRAM_melatonin
107. NGRAM_abdominal^pain
108. NGRAM_gram
109. NGRAM_magnesium
110. FERRITIN_SERUM_PROC_NAME
111. NGRAM_odt
112. NGRAM_odt^ondansetron
113. NGRAM_ambulatory

114. ~~NGRAM_phenergan~~
115. ~~NGRAM_flares~~
116. ~~NGRAM_mouth^needed~~
117. ~~NGRAM_glycol^polyethylene~~
118. ~~NGRAM_polyethylene~~
119. ~~NGRAM_glycol~~
120. ~~NGRAM_psychosis~~
121. ~~NGRAM_urine~~
122. ~~NGRAM_docusate^sodium~~
123. ~~NGRAM_docusate~~
124. ~~ANTIHISTAMINE_-_1ST_GENERATION_-_PHENOTHIAZINES_PHARM_SUBCLASS_NAME~~
125. ~~PROMETHAZINE_HCL_GENERIC_NAME_1~~
126. ~~NGRAM_stomach~~
127. ~~NGRAM_ed~~
128. ~~CREATININEUR(REFERRAL)_COMPONENT_NAME~~
129. ~~MISC_REF_TEST_RESULT_COMPONENT_NAME~~
130. ~~CBC_WITH_DIFFERENTIAL_PROC_NAME~~
131. ~~LAB00681_PROC_CODE~~
132. ~~NGRAM_oral^powder~~
133. ~~NGRAM_powder~~
134. ~~ESSENTIAL_(PRIMARY)_HYPERTENSION_DX_ICD10_NAME~~
135. ~~NGRAM_sulfa~~
136. ~~NGRAM_severe~~
137. ~~NGRAM_penicillins~~
138. ~~NGRAM_gallops~~
139. ~~NGRAM_vicodin~~
140. ~~MISC_REF_TEST_NAME_COMPONENT_NAME~~
141. ~~NGRAM_latex~~
142. ~~NGRAM_zofran~~
143. ~~NGRAM_iv~~
144. ~~NGRAM_discharged~~
145. ~~NGRAM_nausea~~
146. ~~NGRAM_acute~~

~~References~~
